# Molecular Insights into Female Hybrid Sterility in Interspecific Crosses between *Drosophila melanogaster* and *Drosophila simulans*

**DOI:** 10.3390/ijms25115681

**Published:** 2024-05-23

**Authors:** Alexei A. Kotov, Vladimir E. Adashev, Ilia A. Kombarov, Sergei S. Bazylev, Aleksei S. Shatskikh, Ludmila V. Olenina

**Affiliations:** 1 Department of Molecular Mechanisms for Realization of Genetic Information, National Research Centre “Kurchatov Institute”, Moscow 123182, Russia; kotov_alexei@mail.ru (A.A.K.); adashev.vladimir@gmail.com (V.E.A.); bazylevser@gmail.com (S.S.B.); 2 Laboratory of Functional Genomics, Koltzov Institute of Developmental Biology of Russian Academy of Sciences, Moscow 119334, Russia; ilkombarov9192@gmail.com (I.A.K.); shackih@yandex.ru (A.S.S.)

**Keywords:** speciation, female hybrid sterility, oogenesis, piRNA pathway, RDC complex, whole-transcriptome libraries, *Drosophila*

## Abstract

Species of the genus *Drosophila* have served as favorite models in speciation studies; however, genetic factors of interspecific reproductive incompatibility are under-investigated. Here, we performed an analysis of hybrid female sterility by crossing *Drosophila melanogaster* females and *Drosophila simulans* males. Using transcriptomic data analysis and molecular, cellular, and genetic approaches, we analyzed differential gene expression, transposable element (TE) activity, piRNA biogenesis, and functional defects of oogenesis in hybrids. Premature germline stem cell loss was the most prominent defect of oogenesis in hybrid ovaries. Because of the differential expression of genes encoding piRNA pathway components, *rhino* and *deadlock*, the functional RDC^mel^ complex in hybrid ovaries was not assembled. However, the activity of the RDC^sim^ complex was maintained in hybrids independent of the genomic origin of piRNA clusters. Despite the identification of a cohort of overexpressed TEs in hybrid ovaries, we found no evidence that their activity can be considered the main cause of hybrid sterility. We revealed a complicated pattern of Vasa protein expression in the hybrid germline, including partial *AT-chX* piRNA targeting of the *vasa^sim^* allele and a significant zygotic delay in *vasa^mel^* expression. We arrived at the conclusion that the hybrid sterility phenotype was caused by intricate multi-locus differences between the species.

## 1. Introduction

The cessation of gene flow between diverging populations is the necessary condition for a species to split from a common ancestor. Interspecific hybrid sterility is considered a common phenomenon of postzygotic reproductive isolation that significantly contributes to the speciation process [1,2]. Genetic and cellular factors, as well as molecular mechanisms of hybrid sterility, are subjects of considerable interest as they expand our understanding of speciation. Studying *Drosophila* interspecific hybrids allows us to reconstruct the speciation mechanisms and identify hybrid incompatibility factors that maintain postzygotic reproductive isolation between closely related species [3,4,5,6,7,8,9,10,11,12]. 

The fruit fly *Drosophila melanogaster* is completely reproductively isolated from its sibling species of the *Simulans* clade, *Drosophila mauritiana*, *Drosophila sechellia*, and *Drosophila simulans* [13,14]. *D. melanogaster* split from a common ancestor of the *Simulans* clade near three MYA [15,16,17]. However, the genetic basis of hybrid lethality in this case is relatively simple, and the lethality can be overcome by changing a limited number of genomic loci. It was shown that loss-of-function mutations of the X-linked *Hmr* (*Hybrid male rescue*) gene significantly rescue the survival of F1 hybrid progeny from crosses between *D. melanogaster* females and males of the *Simulans* clade [6,7]. A quantitative link was also found between the presence of another X-linked locus, initially called *Zhr* (*Zygotic hybrid rescue*), in the *D. melanogaster* genome and female hybrid lethality in crosses of *D. simulans* mothers and *D. melanogaster* fathers [3,18]. This locus contains the megablock of 359 bp *1.688* satellites in the pericentric heterochromatin of the X chromosome. The presence of this expanded satellite block in hybrid female embryos causes asynchrony of divisions and segregation failures of the paternally inherited X^mel^ chromosome on the syncytial blastoderm stage [19]. The deletion of the bulk of the block or its translocation out of pericentric heterochromatin allows for the rescue of female hybrid lethality [18,19].

However, the molecular and cellular mechanisms underlying hybrid sterility are still largely unknown. They include interchromosomal gene translocations and duplications; genetic divergence at the same locus; dosage imbalance; meiotic drive; unpredictable epistatic interactions among genes that have separately diverged in parental genomes; and unregulated activity of transposons or other harmful elements [11,20,21,22]. The most widely accepted model for the understanding of the genetic basis of hybrid lethality and sterility, the Bateson–Dobzhansky–Muller (BDM) model, is based on the hypothesis that hybrid incompatibility arises from a harmful interaction among functionally diverged genes from the parental species [23,24,25]. This model is supported by genetic data from different organisms, including yeasts, worms, insects, fishes, mice, and plants [26]. Several hybrid sterility loci have been reported to date, but a few are characterized at the molecular level in *Drosophila* [4,10,27]. The search for and identification of hybrid sterility factors is a challenging task in most cases. The background of interspecific hybrids exhibits unique genetic properties that often cannot be predicted from the additive combination of the two parental genomes.

Transposons are ubiquitous genomic components that are able to change their genomic positions and cause insertional mutations, chromosome rearrangements, and double-strand DNA breaks [28,29,30,31]. Recent studies favor the potential role of transposon mobilization in causing interspecific hybrid sterility in *Drosophila* [32,33,34,35]. Transposon silencing in the germline is a critical condition for ensuring genome integrity and transmitting genetic information to the next generation. It is predominantly mediated by the host defense PIWI-interacting RNA (piRNA) pathway with guide piRNAs loaded with PIWI subfamily proteins [36,37,38,39]. Strategic principles of piRNA silencing and biogenesis appear to be conservative across evolution in a wide range of animals [40]. PIWI/ARGONAUTE-clade proteins are central players in the piRNA pathway, carrying out both the generation of piRNAs and the silencing of piRNA complementary targets. Besides PIWI proteins, many other proteins are involved in this process. However, it was found that some genes essential for piRNA biogenesis and transposon silencing in model organisms are poorly conservative and evolve rapidly under positive selection [41,42,43,44].

In the ovaries of *D. melanogaster*, most piRNAs for transposon silencing originate from specialized genome regions called dual-strand or bidirectional piRNA clusters, where both genomic strands are transcribed as long piRNA precursors [37,39]. Transcription of bidirectional piRNA clusters proceeds in a non-canonical mode, being independent of classical promoters of RNA PolII. Transcription of these clusters occurs in the presence of the repressive chromatin mark H3K9me3 [45]. The RDC complex, composed of the nuclear proteins Rhino (Rhi), Deadlock (Del), and Cutoff (Cuff), is shown to be essential for non-canonical transcription of bidirectional piRNA clusters and co-transcriptional processing of piRNA precursors [46,47,48]. Rhi, belonging to the HP1 protein family, recognizes the chromatin of piRNA clusters through H3K9me3 histone modification and attracts the scaffold factor Del. Del recruits Moonshiner, a transcription initiation factor, facilitating transcription in a heterochromatin environment. Del also interacts with Cuff, which prevents transcriptional splicing and termination [46,47,48,49,50]. A bulk of piRNA production in the ovarian germline collapses if any one of the RDC components is lost. Mutations of RDC components cause female sterility and defects of the dorsal–ventral patterning in developing oocytes [46,48,49,51,52].

According to recently published data, all three RDC genes have rapidly evolved under positive selection [42,43,52,53,54]. With the aid of ectopic expression of *D. simulans rhi* and *del* alleles in the *D. melanogaster* mutant background, it was shown that rapid coevolution of *rhi* and *del* genes provides orthologs in the sibling species that do not form functional RDC complexes in the *D. melanogaster* germline [51,52], wherein expression of *rhi* and *del* orthologues from the *D. simulans* genome in the *D. melanogaster* germline causes a disruption of piRNA biogenesis, transposon activation, and subsequent female sterility. Transgenic Rhi^sim^ is not able to interact with Del^mel^, whereas Del^sim^ forms a non-functional complex with Rhi^mel^ in *D. melanogaster* ovaries [51,52]. Also, the *D. simulans cuff* ortholog fails to rescue *D. melanogaster cuff* null mutations and leads to dominant female sterility being overexpressed in *D. melanogaster* ovaries [53]. These findings led to the hypothesis that the adaptive evolution of RDC complex genes may directly contribute to the hybrid sterility between *D. melanogaster* and *D. simulans* [51,52,53]. 

Here, we focus on investigating the female hybrid sterility nature in the crossing between *D. melanogaster* females and *D. simulans* males. Using whole transcriptome sequencing data, in hybrid ovaries, we uncovered differential allele-specific expression of genes encoding components of the piRNA pathway that make up RDC complexes. We found that, in the absence of the RDC^mel^ complex, the RDC^sim^ complex was able to ensure the non-canonical transcription of dual-strand piRNA clusters in hybrid ovaries, independent of their origin. Our results showed that despite the maintenance of adaptive piRNA biogenesis via the ping-pong amplification cycle in hybrid ovaries, the amount of TE-mapped piRNAs was reduced. But we found no evidence that overexpression of the TE cohort in hybrid ovaries can be considered the main cause of hybrid sterility. Rather, we suggest that the observed reproductive incompatibilities were caused by intricate multi-locus genetic and epigenetic differences between the distinct species.

## 2. Results

### 2.1. Hybrid Ovaries Exhibit Multiple Phenotypic Abnormalities of Oogenesis

To analyze the nature of hybrid sterility in crossings between *D. melanogaster* and *D. simulans* flies, we used the *Hmr^2^* line of *D. melanogaster*. It has been shown that the dominant *Hmr* mutation is able to partially rescue the embryonic lethality of hybrid females [7]. We crossed *D. melanogaster Hmr^2^* females and *D. simulans w.501* males to generate F1 hybrid female progeny. The crossing allowed us to obtain adult hybrid females possessing developed ovaries in about one-quarter of cases. For subsequent analyses, we selected the ovaries of hybrid females that had at least several ovarioles containing an array of egg chambers. The ovaries of the rest of the hybrid females had a strongly reduced size and poor morphology and were not used for analysis.

**Figure 1:**
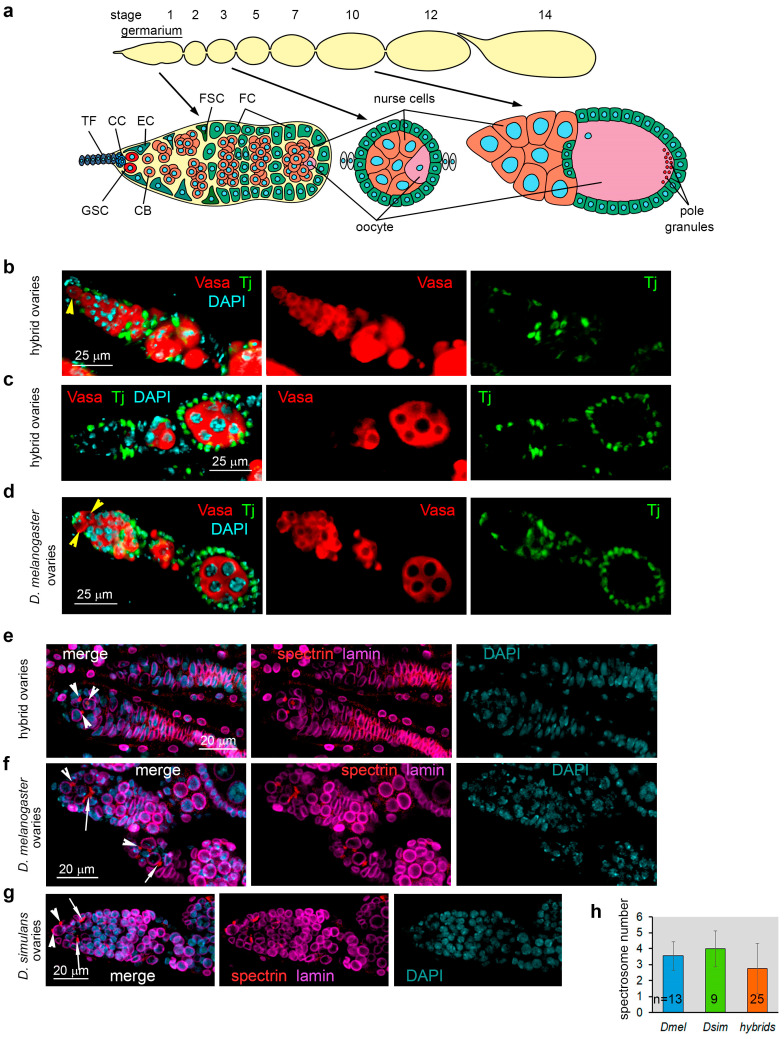
Immunofluorescence analysis of hybrid ovaries. (**a**) The scheme of *Drosophila* oogenesis. Each ovary contains about 16–20 ovarioles. An individual ovariole is shown at the top in a uniform beige color. The stages of oogenesis are indicated at the top of the scheme. At the anterior end of the ovariole, the germarium structure supports 2–3 germline stem cells (GSC) and somatic cells, such as terminal filaments (TFs), cap cells (CCs), and escort cells (ECs). The division of GSCs produces a new GSC and a cystoblast (CB). The germ cell cysts arise from cystoblasts that undergo four synchronous divisions. Each ovariole consists of a string of egg chambers, which comprise an oocyte (light violet) and 15 polyploid nurse cells (red), connected to each other and surrounded by a monolayer of somatic follicle cells (FCs) (green), produced by divisions of follicle stem cells (FSCs, deep green). At the 10/11 stages of oogenesis, the pole plasm is assembled, and pole granules (red dots) are concentrated at the posterior pole of the developing oocyte. (**b**–**d**) Immunofluorescence analysis of the ovaries of hybrid and *D. melanogaster* females. Fixed ovarian preparations were stained with antibodies to Vasa (germ cell marker, red) and Tj (marker of somatic ovarian cells, green), and chromatin was stained with DAPI (blue). All images are oriented with the anterior ends to the left. Germariums of hybrid ovaries can contain GSCs (yellow arrowhead) (**b**), but they often lose them prematurely (**c**). The germarium of *D. melanogaster Hmr^2^* females maintains 2–3 GSCs (yellow arrowheads) (**d**). (**e**–**g**) Analysis of spectrosomes and fusomes in the germariums of hybrid ovaries (**e**), *D. melanogaster* ovaries (**f**), and *D. simulans* ovaries (**g**). Fixed ovarian preparations were stained with antibodies to a-spectrin (red) and Lamin (a marker of nuclear envelopes, violet), and chromatin was stained with DAPI (blue). White arrowheads indicate spectrosomes, and white arrows indicate fusomes. (**h**) Number of spectrosomes per germarium in parental species and hybrids (in the case of germ cell-positive germariums). Data are represented as average ± SD (standard deviation), and the numbers of analyzed germariums (n) are indicated on the graph.

Compared with other developmental processes, fly oogenesis can be examined in detail [55]. The ovary of *D. melanogaster* flies in the norm contains about 16–20 individual ovarioles [56]. At the anterior end of the ovariole, the germarium structure supports two to three germline stem cells (GSCs) and somatic ovarian cells (Figure 1a). The self-renewal division of GSC produces a new GSC and a cystoblast (CB). The germ cell cysts arise from CBs that undergo four synchronous mitotic divisions. Each ovariole consists of an array of egg chambers, which comprise an oocyte and 15 nurse cells, interconnected to each other and surrounded by a monolayer of somatic follicle cells produced by divisions of follicle stem cells. At the 10/11 stages of oogenesis, pole granules are assembled and concentrated at the posterior pole of the developing oocyte, allowing subsequent primordial germ cell specification in embryos. To study the ovarian phenotypes involved in hybrid sterility, we dissected ovaries from adult females (3–6 days after eclosion) obtained from interspecific crosses and parental species. We performed a phenotypic analysis of the developed hybrid and parental ovaries using immunostaining and confocal microscopy. For germ cell identification, we used rabbit polyclonal anti-Vasa antibodies. Our previous testing of these antibodies showed that they recognize both Vasa^sim^ and Vasa^mel^ proteins in Western blots (Figure S1a). Analyzing fixed ovarian preparations, we found that the majority of germariums of hybrid ovaries did not contain germ cells. However, at least 14.1 ± 8.4% of the ovarioles in the hybrid ovaries (n = 30) contained one to multiply Vasa-positive germ cells (Figure 1b,c and Figure S1b). The ovaries of *D. melanogaster Hmr^2^* females exhibit a wild-type phenotype, maintaining germ cells throughout oogenesis (Figure 1d).

In the *Drosophila* ovary, from two to three GSCs and their immediate progeny, CBs in the germarium can be identified by the presence of a small spherical structure, the spectrosome, marked by α-spectrin, whereas germ cell cysts subjected to mitotic divisions can be recognized by the presence of α-spectrin-positive branched fusomes [57,58]. We detected germline content, including GSCs at the apical tip, in the germariums of individual ovarioles of hybrid ovaries (Figure 1b). In these germ cell-positive germariums, we also detected the presence of spectrosomes, markers of GSCs and CBs, on average 2.76 ± 1.56 per germarium (Figure 1e (white arrowheads),h). The number of spectrosomes was not significantly different from that of parental species, which contained from 3.54 to 4.0 spectrosome-positive germ cells per germarium, as expected (Figure 1f,g (white arrowheads),h). Thus, our analysis suggests that primordial germ cells in hybrid embryos are able to migrate through the endodermal epithelium into the mesoderm and coalesce with the somatic gonadal precursor cells to form ovaries. However, we found that branched fusomes, markers of mitotically active germ cells, were detected only in 36% of the germ cell-positive germariums of hybrid ovaries, indicating a frequent lack of germ cells at the stage of mitotic amplification.

**Figure 2:**
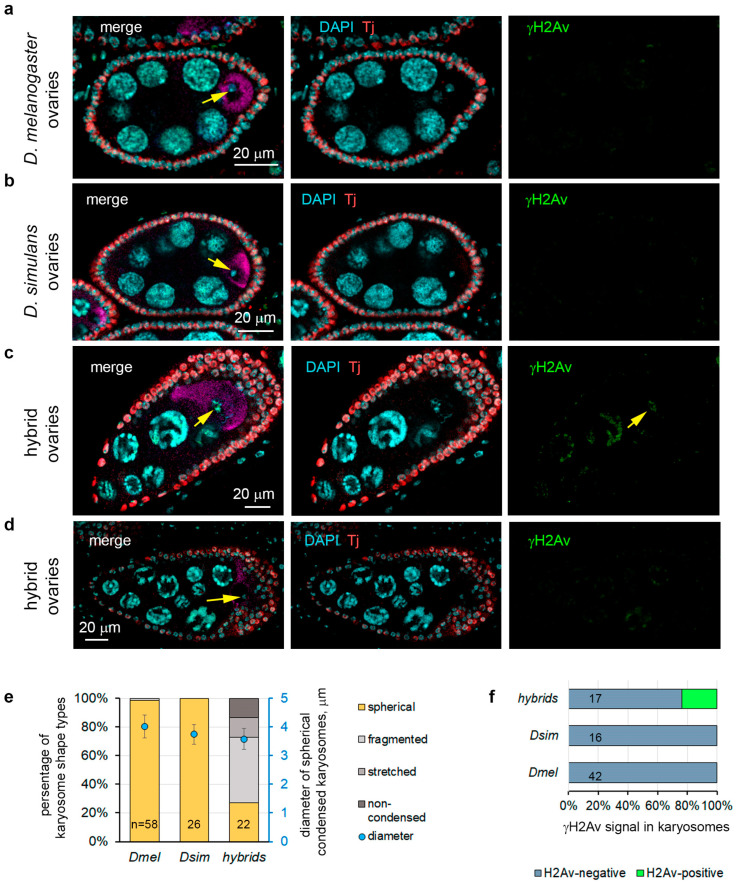
Oocyte specification in hybrid ovaries. (**a**–**d**) Immunofluorescence analysis of the ovaries of parental females (**a**,**b**) and hybrids (**c**,**d**). Fixed ovarian preparations were stained with antibodies to Orb (oocyte marker, violet), Tj (marker of somatic ovarian cells, red), and γ-H2AV (marker of DSBs, green), and chromatin was stained with DAPI (blue). All images are oriented with the anterior end to the left. The karyosomes are indicated by yellow arrows. (**c**) Karyosomes are often fragmented in the egg chambers of hybrid ovaries. (**e**) Distribution of wild-type (compact spherical) and defected (fragmented, stretched, and non-condensed) karyosomes in hybrid and parental oocytes. Data are represented as percentages for karyosome shape types (bars) and as average ± SD for spherical karyosome diameters (blue dots). n, the number of karyosomes examined for each fly line. (**f**) Analysis of γ-H2AV-positive karyosomes in hybrid ovaries and ovaries of parental species. The data are represented as percentages of γ-H2AV-positive and -negative karyosomes. No karyosomes with DSBs in parental species oocytes were found. The numbers of karyosomes examined for each fly line are indicated on the graph.

We used antibodies to the Orb protein to mark oocytes [59] at the basal end of 16-cell cysts and found clear Orb staining in the egg chambers in the ovaries of *D. melanogaster* and *D. simulans* females (Figure 2a,b). We observed a close to correct development of egg chambers with specifications of oocytes in more than half of the developed egg chambers of hybrid ovaries (Figure 2c,d). However, in other cases, we did not find Orb staining in the egg chambers, which indicated failures of oocyte specification in hybrids. In normal conditions, the DNA of developing oocytes is typically packed into a spherical condensed structure called the karyosome [60]. Using DAPI chromatin staining, we analyzed the karyosome morphology in hybrid and parental ovaries. Whereas in *D. melanogaster* and *D. simulans* egg chambers (from stages 3 to 9), 98.3–100% of karyosomes maintain the spherical form with an average diameter of 4.01 µm and 3.74 µm (Figure 2a,b,e), in hybrid ovaries, the karyosomes exhibit packing defects, including fragmented, stretched, and non-condensed shapes in 72.7% of the cases (Figure 2c,e). Only 27.3% of the karyosomes of hybrids have a wild-type spherical shape and size with an average diameter of 3.58 ± 0.37 µm (Figure 2d,e).

We found that the oogenesis of hybrid females proceeds with multiple disturbances. The most common among them is a loss of GSCs, manifested by germariums containing only Tj-positive somatic cells (Figure S1b) or the presence of differentiating germ cells in the apical part of the germarium, where GSCs should normally be located (Figure S1c). The disorders we identified also include an absence of germ cells at the stage of mitotic amplification in germarium (mitotic arrest of self-renewing GSC division); egg chambers with cysts that are not passing through four mitotic divisions and contain less than 16 germ cells; an irregularity of egg chamber development within the same ovariole; and premature degeneration of the egg chambers. Most hybrid females were not able to carry out oogenesis until the late stages. Only a small cohort of hybrid females had perfectly developed ovaries and maintained oogenesis throughout the late stages up to the maturation of oocytes. We found hybrid oocytes in 69.1% of cases (n = 68) with fused dorsal appendages, indicating a disruption of epidermal growth factor signaling and defects of the egg dorsoventral patterning (Figure S2). Taken together, we revealed that hybrid females from crossing *D. melanogaster* and *D. simulans* possessed a wide range of oogenesis defects and were completely infertile.

### 2.2. Differential Gene Expression in Hybrid Ovaries

**Figure 3:**
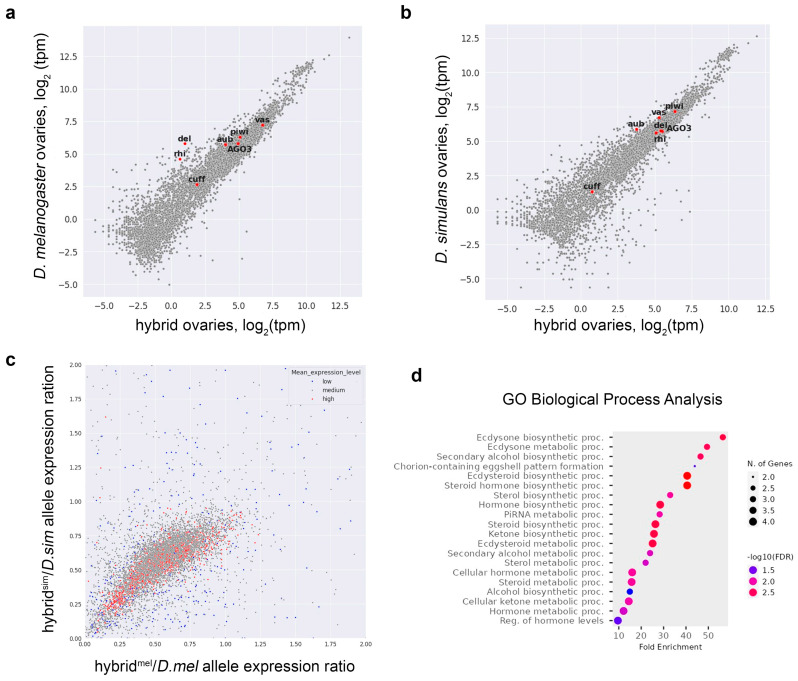
Differential gene expression between hybrid ovaries and ovaries of their respective parental species. (**a**,**b**) Scatterplots represent species-specific differential gene expression (in transcript per million (tpm) number) in the ovaries between interspecific hybrids and parental species, *D. melanogaster* (**a**) and *D. simulans* (**b**). See Tables S1 and S2 for DEseq data. Key genes involved in the piRNA pathway are marked as red dots on both scatterplots. *Rhi^mel^* and *del^mel^* occupy off-diagonal positions (**a**), indicating their differential allele-specific expression in hybrids. (**c**) Analysis of the expression level of *D. melanogaster* and *D. simulans* inherited genes in the hybrid ovaries relative to their expression in the ovaries of the parental species. The scatterplot represents a comparison of hybrid/parent expression ratios for species-specific orthologues. The dot color indicates the average level of gene expression in the ovaries parents: low (blue): log_2_(cpm) ≤ 5; medium (gray): 5 < log_2_(cpm) ≤ 12; high (red): 12 < log_2_(cpm). See Figure S3 and Table S3 for the data. (**d**) Dotplot for the top 20 categories of GO Biological Processes for down-regulated *D. melanogaster* alleles in hybrid ovaries. The analysis was performed using ShiniGO 0.80 software with data obtained from DEseq analysis. See Table S4 for detailed annotations.

To study oogenesis in the hybrid ovaries in more detail, we prepared and sequenced whole-transcriptome libraries of mRNAs isolated from the ovaries of interspecific hybrids and parental species. Using the RNA-seq data, we perform the analysis of differential gene expression (DEseq) between *D. melanogaster* and hybrid ovaries (Figure 3a, Table S1) and between *D. simulans* and hybrid ovaries (Figure 3b, Table S2). The scatterplots (Figure 3a,b) display a correlation at the transcriptional level for species-specific alleles between the hybrid ovaries and parent species. According to the data, most of the analyzed genes have no significant differences in their expression in hybrid ovaries compared to parents (Pearson correlation coefficients of 0.94 and 0.93 for *D. mel*-hybrid and *D. sim*-hybrid comparisons, respectively).

Using the annotation sets of protein-coding genes and RNAseq data, we performed a comparative analysis of the expression levels of *D. melanogaster* and *D. simulans* orthologous alleles in the ovaries of hybrids relative to their average expression levels in the ovaries of the parental species (Figure 3c and Figure S3; Table S3). The scatterplot represents ratios of gene expression in the hybrid ovaries compared to the parental ones (Figure 3c). According to hybrid/parent expression ratios for species-specific orthologues, hybrid ovaries exhibit the expected expression level of 60.3% of the cases for both orthologous parental alleles (Figure S3). In 15.2% of the cases in hybrid ovaries, only one allele was found to be overexpressed with the maintenance of the expected expression level of the other allele. In only 9.8% of the cases, both parental alleles in hybrids were overexpressed. In 5.2% of the cases, both alleles were found to be underexpressed (Figure S3).

Next, we selected cohorts of genes with tenfold up- and down-expression differences in hybrid ovaries and performed GO analysis using ShiniGO 0.80 software [61]. The GO Biological Process search of the selected genes of hybrid ovaries inherited from the *D. melanogaster* genome with tenfold and more underexpression revealed the enrichment of small sets of genes involved in the piRNA pathway and also in a group of pathways associated with ecdysone and other steroid hormone metabolic processes and regulation (Figure 3d). Only seven genes were included in the top 20 GO Biological Process categories, among them *shadow* (*sad*), *disembodied* (*dib*), *shroud* (*sro*), and *GstE14*, which were shared in steroid hormone-related categories and the piRNA pathway genes including *rhino* (*rhi*), *deadlock* (*del*), and *Oxpecker* (*Oxp*) (Table S4). For the cohort of up-regulated *D. melanogaster* genes in the hybrid ovaries, we did not find any enrichment for certain GO categories (not shown).

### 2.3. Expression of Key piRNA Pathway Factors in Hybrid Ovaries

**Figure 4:**
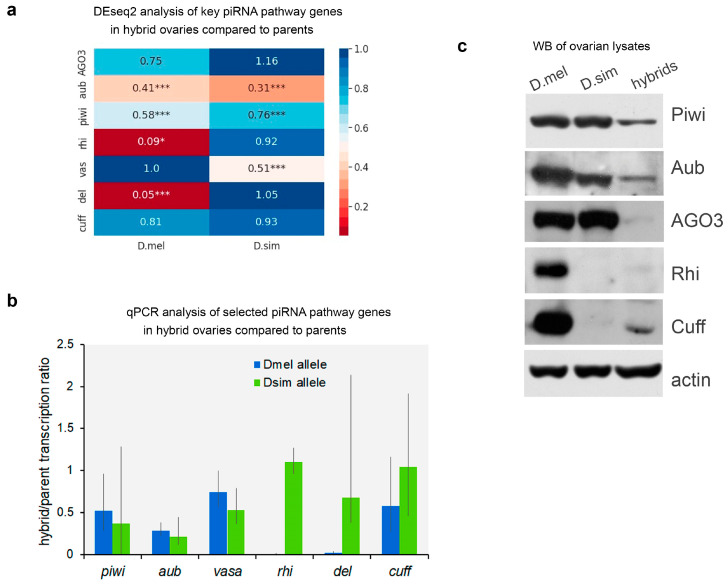
Expression of piRNA pathway factors in the ovaries of hybrids. (**a**) Heat map of allele-specific expression of key piRNA pathway genes in the ovaries of hybrids. Differential expression analysis was performed using DESeq2. The fold change ratios of *D. melanogaster* (D.mel) and *D. simulans* (D.sim) allele expression in hybrids versus parental species are shown. Statistically significant differences are indicated using an adjusted *p*-value. One asterisk (*) indicates p^adj^ < 0.05. Three asterisks (***) indicate p^adj^ < 0.001. (**b**) RT-qPCR analysis of piRNA pathway gene expression in the ovaries of hybrids compared to parental species, *D. melanogaster* and *D. simulans*. Fold change ratios of the average expression level in hybrids relative to the parental lines are shown for each species-specific allele. Error bars represent 95% confidence intervals for the data from three independent experiments. *Rhi^mel^* and *del^mel^* alleles were not expressed in hybrid ovaries. The expression levels of mRNAs are normalized to *rp49* transcripts. See also Figure S4 for data presentation. (**c**) Western blot analysis of piRNA pathway proteins in hybrid ovaries and parental lines. Anti-actin antibodies were used for the loading control. Representative data from at least three independent experiments are shown.

Considering that the piRNA pathway is a critical mechanism involved in maintaining oogenesis in *Drosophila* [39,62], we focused on the examination of the genes involved in piRNA biogenesis. Comparative analysis of gene expression in the ovaries of hybrids revealed striking differences in the *rhi* and *del* alleles from parental species. Whereas the expression of *rhi* and *del* inherited from *D. melanogaster* was strongly suppressed, the corresponding *D. simulans* alleles were supported on the anticipated expression level in the hybrid ovaries (Figure 3a,b, red dots, and Figure 4a). Our DEseq analysis also showed that expression of both species-specific alleles of *aubergine* (*aub*) was significantly decreased in hybrids, and the *D. simulans* allele of *vasa* exhibited half the predicted expression level (Figure 4a). We also found significant differences in *piwi* expression levels in hybrid ovaries compared with parental flies. In total, these data lead to the proposal that piRNA biogenesis and piRNA-mediated silencing may be partially compromised in the oogenesis of hybrids.

We confirmed the results of DEseq for selected piRNA pathway-related genes by RT-qPCR analysis of ovarian transcripts with species-specific primers (Figure 4b and Figure S4). According to RT-qPCR data, the *D. melanogaster* inherited alleles of *rhi* and *del* were not practically expressed in hybrid ovaries. However, the *rhi* and *del* alleles originating from the *D. simulans* genome exhibited expression above the expected half dose. Taken together, both DEseq and RT-qPCR analyses indicated that at least two of the three components of the RDC complex encoded by *D. melanogaster* alleles are absent in the hybrid ovaries.

We performed a Western blot analysis of ovarian lysates (Figure 4c) with antibodies produced to proteins from *D. melanogaster*, the specificity of which did not always allow us to recognize orthologues from *D. simulans*. We found that PIWI subfamily proteins of both species, Piwi, Aub, and AGO3, were recognized by antibodies with similar efficiency. We revealed that PIWI proteins were expressed in hybrid ovaries at a lower level than in the parents. We expectedly detected an absence of the Rhi^mel^ protein in hybrid ovaries, in accordance with data about its transcriptional level. We found only a very low level of the Cuff^mel^ protein in hybrids, despite maintaining *cuff^mel^* transcription. However, we could not detect the presence of the Rhi^sim^ or Cuff^sim^ proteins in hybrid ovaries because they were not recognized by the available antibodies. In general, these data confirm that the RDC^mel^ complex is not formed in hybrid ovaries.

According to previously published data, the Rhi and Del proteins in *D. melanogaster* and *D. simulans* species diverged significantly under positive selection and are not able to form functional complexes with orthologues from sibling species [51,52,63]. This circumstance could presumably contribute to disrupting the functioning of the piRNA pathway, mobilization of transposons in the germline, and interspecific hybrid sterility. However, our results indicate that the RDC complex encoded by *D. melanogaster* alleles is not assembled in the ovarian germline of *D. mel/D. sim* interspecific hybrids. 

### 2.4. RDC^sim^ Complex Acts in Hybrid Ovaries

**Figure 5:**
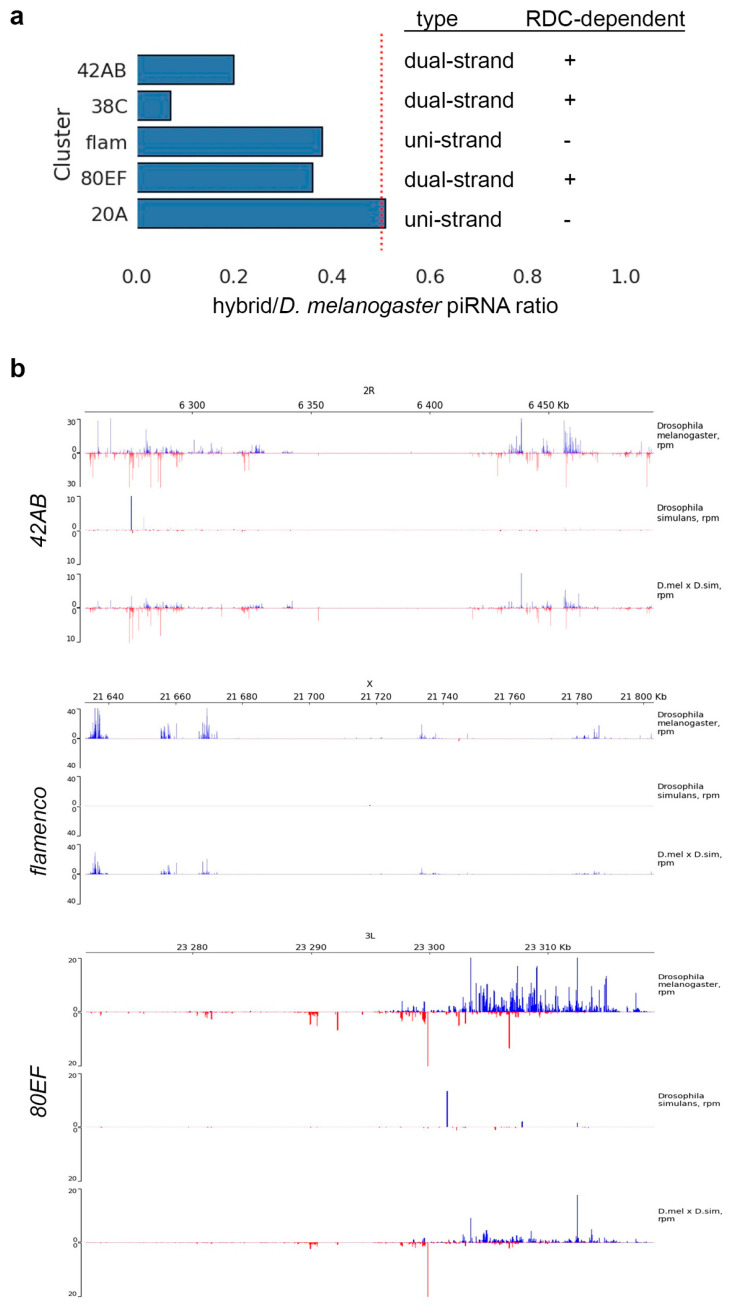
Analysis of piRNAs mapped to piRNA clusters. (**a**) Uniquely mapped cluster-derived piRNAs in hybrid ovaries relative to the *D. melanogaster* level. Data for the top five piRNA clusters of *D. melanogaster* ovaries are presented as the ratio hybrid/*D. melanogaster* piRNA counts. The red dotted line shows the expected relative level of piRNAs for hybrid ovaries. Both RDC-dependent and RDC-independent piRNA clusters maintain activity in hybrids. (**b**) piRNA genome distribution analysis across *42AB*, *flamenco* and *80EF* clusters. Blue peaks indicate mapped antisense piRNAs, and red peaks indicate sense piRNAs.

We asked whether the RDC^sim^ complex functions in the hybrid genome environment to maintain piRNA cluster transcription. Two main types of piRNA clusters are described in *Drosophila*. There are uni-strand clusters transcribed from a single genomic strand that produce piRNAs mapping only to this strand and dual-strand clusters transcribed in both directions [37,39,47,64]. Because the RDC complex is known to be responsible for the transcription of dual-strand clusters in the *Drosophila* germline [46,47,48,49], we decided to analyze the piRNA production of these clusters in hybrids. For this analysis, we generated and sequenced libraries of small RNAs from hybrid and parental ovaries. According to published data, among the top five piRNA clusters in *D. melanogaster* ovaries, *42AB*, *80EF*, and *38C* are RDC-dependent dual-strand clusters, whereas *flamenco* and *20A* are canonical uni-strand clusters [39,47,64]. Because of the repetitive nature of most piRNA clusters and the unambiguous species-specific piRNA definition, cluster activity can be generally identified by the count of unique piRNAs mapping to parental genomes. Our analysis revealed that both types of major clusters inherited from the *D. melanogaster* genome, RDC-dependent and RDC-independent ones, were still active as sources of piRNAs in hybrid ovaries (Figure 5). These results indicate that despite lacking a functional RDC^mel^ complex, the RDC^sim^ complex is able to recognize and license non-canonical transcription of dual-strand clusters in the hybrid genome background, independent of cluster origin.

### 2.5. Transposon Expression in Hybrid Ovaries

**Figure 6:**
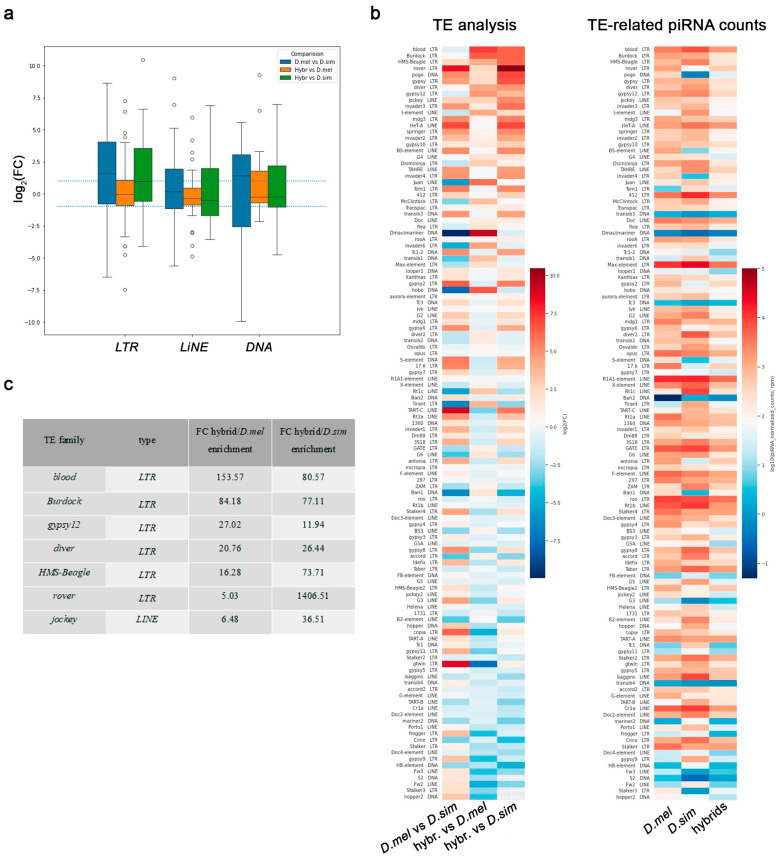
TE expression in hybrid ovaries. (**a**). DEseq analysis of the main TE types (*LTR*, *LINE*, and *DNA*) between the ovaries of parental species and between the ovaries of hybrids and parents is presented as log_2_(fold change ratio). The dotted line represents log_2_(fold change) > |1| and adjusted *p*-value < 0.05. (**b**) Left: heat map presenting comparative TE expression analysis between the ovaries of hybrids and parental species. The color indicates log_2_(fold change ratio). Right: heat map presenting TE-related piRNA counts in hybrids and parental species as log_10_(normalized counts). (**c**) Seven TE families that demonstrated overexpression by 5 or more fold changes (FC, p^adj^ < 0.05) in hybrid ovaries compared with parental species.

The piRNAs produced by major ovarian piRNA clusters are mainly directed at preventing TE propagation at the transcriptional and post-transcriptional levels [37,38,39]. We carried out a comparative DE analysis of transposon transcripts using our polyA-selected RNAseq library data. DEseq analysis for 179 fly transposon families (according to the resource https://github.com/bergmanlab/drosophila-transposons/ (accessed on 30 August 2021)) in the ovaries of parental species showed that only 120 of them are expressed in at least one of the species. Comparative DEseq analysis of the main types of TEs (*LTR* (*Long Terminal Repeats*), *LINE* (*Long Interspersed Nuclear Element*), and *DNA* ones) between the ovaries of parental species and between the ovaries of hybrids and parents is presented in Figure 6a. According to the data, most TEs did not exhibit a significant increase in their transcription in hybrid ovaries. We found that 32 families showed a significant increase in their expression level more than fivefold for *D. melanogaster* ovaries versus *D. simulans* ovaries, and 15 families were overexpressed for *D. simulans* ovaries versus *D. melanogaster* with p^adj^ < 0.05 (Figure 6b). Comparative DEseq analysis between hybrids and parental lines revealed that 23 (26.7%) and 15 (12.5%) families of transposons were found to be overexpressed in hybrid ovaries by five or more times relative to *D. simulans* and *D. melanogaster*, respectively (Figure 6b). Note that 19 of these 23 TE families in hybrid ovaries were overexpressed in *D. melanogaster* ovaries versus *D. simulans*, and 6 of 15 TEs were overexpressed in *D. simulans* versus *D. melanogaster*. In sum, comparisons of hybrid ovaries with parental ones revealed increased DE for 31 (25.8%) transposon families compared with at least one of the parental species. At the same time, only seven TE families (5.83%) demonstrated overexpression by five or more times (p^adj^ < 0.05) in hybrid ovaries compared with both parental species. There are six transposons related to *LTR*, including *blood*, *Burdock*, *gypsy12*, *diver*, *HMS-Beagle*, and *rover*, and one, *jockey*, related to *LINE* (Figure 6b,c). Note that only two TEs, *blood* and *Burdock*, exhibited more than 50-fold overexpression in hybrid ovaries compared with both parental species. Compared with that, 17 TE families were found to exhibit more than 50-fold derepression in *D. melanogaster* ovaries of *rhi* mutants [64].

### 2.6. Biogenesis of TE-Related piRNAs Is Maintained in Hybrid Ovaries

**Figure 7:**
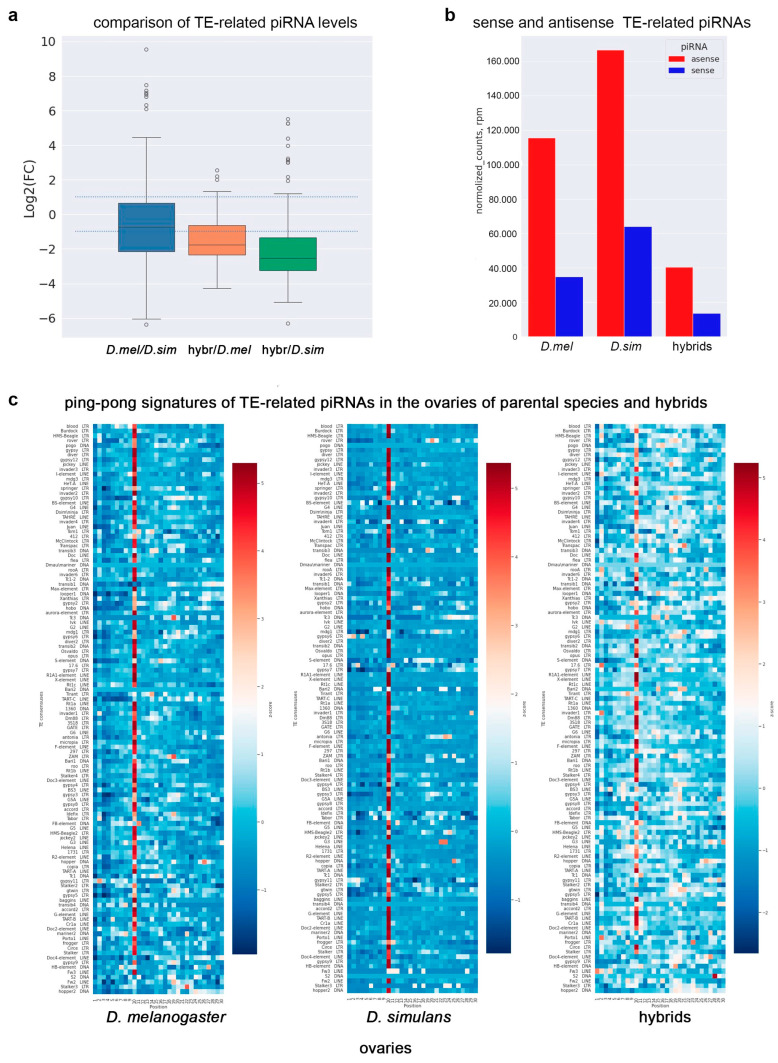
TE-related piRNA production in hybrid ovaries. (**a**) Comparative analysis of total presented as log_2_(fold change ratio). Dotted lines represent log_2_(fold change) > |1|. (**b**) The amount of TE-related sense and antisense piRNA normalized reads (in rpm) expressed in the ovaries of *D. melanogaster*, *D. simulans*, and hybrids. (**c**) Heat map presentation comparing ping-pong signatures (z_10_-score values) for TE-related piRNA levels in the ovaries of *D. melanogaster*, *D. simulans*, and hybrids expressed as the number of overlapping pairs (5′-to-5′ distances between complementary piRNA pairs) normalized by the number of piRNAs. The ping-pong z-score was calculated for 1–30 nt positions of piRNAs. The deep red color highlights TEs with stronger ping-pong signatures.

Could the limited number of TEs that are significantly overexpressed in hybrid ovaries be responsible for the disruption of oogenesis and female hybrid sterility? To evaluate this question, we first performed a comparative analysis of TE-derived piRNAs from the ovaries of hybrids and parental species. Indeed, we determined that the total TE-related piRNA pool in hybrid ovaries was reduced compared with the ovaries of both parental species (Figure 6b and Figure 7a,b). The normalized count of total TE-related sense and antisense piRNAs in hybrid ovaries was found to be three- to fivefold less than in parental species (Figure 7b). It is known that the ping-pong amplification process is the distinguishing mechanism of piRNA biogenesis in the germline [37,38], adaptively ensuring effective TE silencing. PIWI protein-mediated slicing of long piRNA precursors provides secondary sense piRNAs that typically overlap by 5′-end 10 nt with antisense piRNAs. The prevalence of 10 nt overlapping sense–antisense piRNA pairs is considered a signature of the ping-pong mechanism, expressed as a z10 score. Our analysis revealed ping-pong signatures for TE-related piRNAs in the ovaries of both parental species (Figure 7c), consistent with previously published data [65]. We also found clear ping-pong signatures for most TE families in the ovaries of interspecific hybrids, indicating that the ping-pong amplification process is active in hybrids (Figure 7c).

Compared with the ovaries of parental females, ping-pong signatures in hybrid ovaries were significantly more weakly expressed for *blood*, *HMS-Beagle*, *rover*, and *jockey* (Figure 7c). We estimated comparative piRNA silencing potential according to [64] for all seven overexpressed TEs, considering it true when there was more than 100 rpm of antisense TE-mapped piRNAs (0–3 mm) (Table 1). Overexpression in hybrid ovaries for all seven TEs was accompanied by piRNA loss by at least 2.6- to 13.4-fold compared with the ovaries of *D. melanogaster* females. We observed a fivefold reduction in antisense piRNAs compared with both parental lines for three of these seven TEs and to at least one parental line for six of these seven TEs. Only *rover* demonstrated a less dramatic decrease in piRNA levels. In addition, only piRNAs from three of the seven transposons (*blood*, *gypsy12*, and *rover*) exceeded the 100 rpm threshold; for the other four overexpressed TEs (*Burdock*, *diver*, *HMS-Beagle*, and *jockey*), piRNA silencing potential in hybrid ovaries was not found to be enough.

According to this observation, we asked if this meant that overexpression of these TEs leads to a high frequency of hybrid genome invasions and subsequent disruption of oogenesis. It is known that retrotransposons rarely mobilize in germline stem cells; however, they selectively mobilize into the DNA of developing oocytes in egg chambers [31,66]. The derepression of retrotransposons and *LINEs* leads to the delivery of their transcripts from nurse cells via microtubule-mediated transport in the oocyte for integration by a “cut-and-paste mechanism” into various loci of its genome. It induces multiple DNA double-strand breaks (DSBs) in the chromatin of developing oocytes, causing severe defects of genome integrity and replication stress, as it has been detected in *D. melanogaster* when the piRNA pathway is disrupted [31]. Retrotransposon integration events can be detected by immunostaining a marker of DSBs, a phosphorylated version of the histone variant (γ-H2Av). γ-H2Av signals are also observed in the norm in mitotic germ cells of the germarium. Using antibodies to γ-H2Av, we found that in hybrid ovaries, this signal is more barely detected in the germarium (16.13% of the ovaries in the preparations possessed at least one γ-H2AV-positive germarium, n = 31), compared to the ovaries of *D. melanogaster* (50.0% of the ovaries possessed at least one γ-H2AV-positive germarium, n = 22) and *D. simulans* (28.0% of the ovaries possessed at least one γ-H2AV-positive germarium, n = 25). In the egg chambers of parental species, from 98.3 to 100% of developing oocytes (stages 3–9) had condensed karyosome shape and size, as mentioned above (Figure 2a,b,e). We found that none of the examined developing oocytes from parental species were positive for γ-H2Av staining (n = 42 for *D. melanogaster* and n = 16 for *D. simulans*) (Figure 2a,b,f). In hybrid ovaries, we often revealed either stretched or fragmented karyosomes (Figure 2c–e), indicating catastrophic genome failures in developing oocytes. However, we detected γ-H2AV-positive karyosomes in hybrid ovaries only in 23.5% of the cases (n = 17) (Figure 2c,f). This is inconsistent with massive retrotransposon invasions as a major cause of hybrid sterility. These results indicate that hybrid oogenesis defects are not always associated with increased activity of retrotransposons in ovarian nurse cells and the integration of their transcripts into the genome of the developing oocyte.

### 2.7. Vasa Expression in Hybrid Ovaries

Taking into account the results of DEseq analysis of key piRNA pathway genes (Figure 4a), we dissected a pattern of expression of RNA helicase Vasa in hybrid ovaries. Earlier, we showed that multiple *AT-chX* repeats harbored on the X chromosome of *D. melanogaster* generate a great number of piRNAs with homology to the sequence of the fourth and fifth exons of *vasa* [11]. *AT-chX* piRNAs are expressed both in the testes and ovarian germline; however, sequence identity between *AT-chX piRNAs* and *vasa* appears to be insufficient for repression of *vasa* in the gonads of *D. melanogaster* [11,64]. In contrast, strong silencing has been observed earlier for the *vasa^mau^* allele in the testes of interspecific hybrids *D. melanogaster/D. mauritiana*, owing to a high complementarity between the target and *AT-chX* piRNAs, whereas the *vasa^mel^* allele does not undergo repression [11]. High sequence identity (more than 90%) between *D. melanogaster AT-chX* repeats and mRNA sequences of *vasa* of all three species of the *Simulans* clade [11] allows us to propose similar piRNA-dependent repression of the *vasa^sim^* allele in the ovaries of *D. mel/D. sim* hybrids.

**Figure 8:**
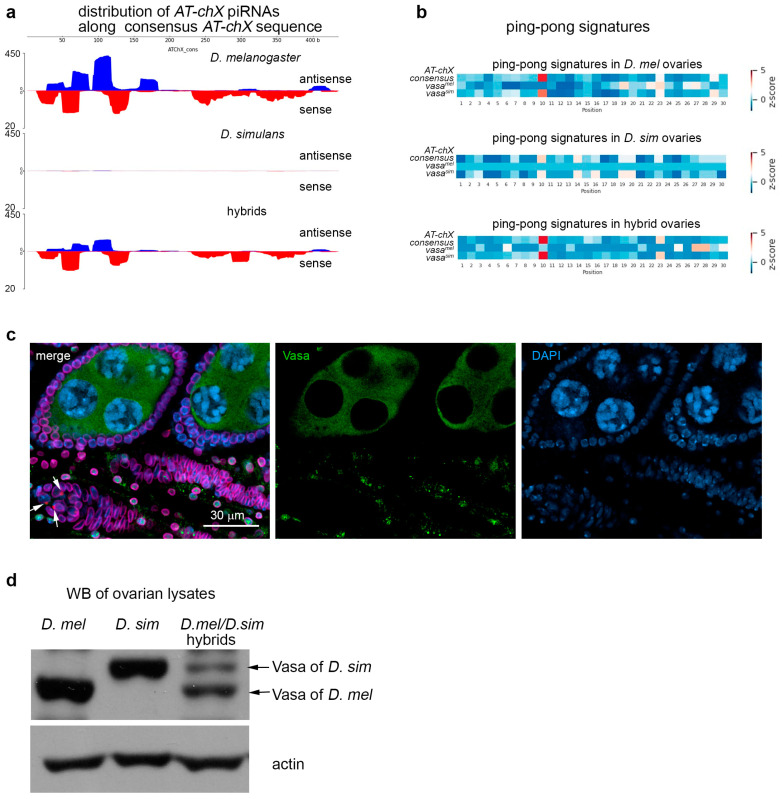
Patterns of Vasa protein expression in hybrid ovaries. (**a**) *AT-chX* piRNA distribution analysis along *AT-chX* consensus permitting 0–3 mismatches. Blue peaks indicate mapped antisense piRNAs, and red peaks indicate sense piRNAs (in relation to *vasa* transcripts). Whereas *AT-chX* piRNAs are absent in *D. simulans* ovaries, in hybrid ovaries and in *D. melanogaster*, they are detected. (**b**) Heat map presentation of ping-pong signatures of piRNAs mapped to species-specific alleles *vasa* and *AT-chX* consensus in gonads of hybrids *D. mel/D. sim* and parental species, *D. mel* and *D. sim*. Fractions of 23–29 nt piRNAs from the ovaries of hybrids and parental lines were mapped to the *AT-chX* consensus and species-specific transcripts of *vasa* genes *D. melanogaster* and *D. simulans* with 0–3 mismatches. Enrichment of ping-pong pairs mapped on *AT-chX* and *vasa^sim^* but not *vasa^mel^* is found in *D. melanogaster* and hybrid ovaries. (**c**) Immunofluorescence analysis of the ovaries of hybrid females. Fixed ovarian preparations were stained with antibodies to Lamin (nuclear envelope marker, violet), Vasa (rat monoclonal antibody, green), and α-spectrin (marker of spectrosomes, red), and chromatin was stained with DAPI (blue). The specrtosomes are indicated by white arrows. The Vasa^mel^ signal is not detected in the early germ cell, including GSCs (marked by spectrosome structures). Vasa^mel^ expression starts in egg chambers after stage 3. (**d**) Western blot analysis of Vasa proteins in the ovaries of hybrid and parental species using anti-Vasa rabbit polyclonal antibodies. Vasa^mel^ and Vasa^sim^ exhibit different electrophoretic mobilities. In total ovarian lysates of hybrids, the signal of Vasa^sim^ is significantly weaker than Vasa^mel^. Anti-actin antibodies were used for loading control. Representative data from at least three independent experiments are shown.

We revealed that the *AT-chX* piRNA cluster was active in the ovaries of *D. mel/D. sim* hybrids, generating both sense and antisense piRNAs related to *vasa* transcripts (Figure 8a). Analysis of overlapping piRNA pairs in the ovaries of hybrids and parental species showed that ping-pong signatures were clearly observed only for *AT-chX* and *vasa^sim^*-mapped piRNA pairs but not for *AT-chX* and *vasa^mel^* (Figure 8b). We asked if the presence of high complementary piRNAs leads to specific silencing of *vasa^sim^* in the background of hybrid ovaries. 

To study Vasa protein expression throughout oogenesis, we used anti-Vasa antibodies with different specificities. We found that rat monoclonal antibodies raised against Vasa (obtained from DSHB) recognize only the Vasa^mel^ protein, but rabbit polyclonal antibodies recognize both Vasa^sim^ and Vasa^mel^ in the immunostaining of fixed ovarian preparations and Western blot analysis (Figure S1). Using confocal microscopy and taking advantage of different antibody specificities, we showed that only the Vasa^sim^ protein was definitely expressed in the hybrid ovaries at the early stages of oogenesis, in germariums, and at stages 2 and 3. Whereas Vasa^mel^ constantly started to be expressed only at later stages if the process of oogenesis in hybrid ovaries reached them (Figure 8c). Thus, we additionally detected a significant delay in the zygotic expression of the *vasa^mel^* allele in the hybrid background, indicating that only the *vasa^sim^* allele is expressed during postzygotic gonad development and early oogenesis.

As we mentioned above, the *vasa^sim^* allele exhibited a half-expected expression level in hybrid ovaries according to DEseq analysis (Figure 4a). Using Western blot analysis, we found that the comparative level of the Vasa^sim^ protein in hybrid ovaries was reproducible three- to fivefold lower than Vasa^mel^ (Figure 8d). These results point in favor of partial piRNA-mediated repression of the *vasa^sim^* allele. Taking into account a delay in the zygotic expression of the *vasa^mel^* allele, we concluded that during gonad development and early stages of oogenesis in hybrid females, the expression of Vasa was significantly lowered.

## 3. Discussion

Interspecific hybrid sterility emerges as a consequence of the accumulation of genetic differences among isolated populations of the ancient precursor [2,20]. The study of interspecific hybrid incompatibilities allows for the reconstruction of speciation mechanisms and the identification of factors that maintain postzygotic reproductive isolation between closely related species. Here, we look into the nature of female hybrid sterility in crossing between *D. melanogaster* females and *D. simulans* males. With the aid of phenotypic analysis of hybrid ovaries, we revealed that the oogenesis of hybrids proceeded with multiple disturbances (Figure 1, Figure 2 and Figure S1), predominantly including a premature loss of germline stem cells (GSCs). However, the oogenesis in some hybrid ovaries proceeded up to late stages, allowing us to perform cellular and molecular analysis.

A disruption of the piRNA pathway leads to severe developmental defects in the ovaries of *D. melanogaster*, causing violations in GSC maintenance, genome damage, and subsequent female fertility [31,39,67]. Taking advantage of whole-transcriptome analysis, we found that several piRNA pathway genes exhibited differential allele expression in hybrid ovaries and could potentially influence transcriptional and post-transcriptional piRNA silencing mechanisms (Figure 4a). As has been proposed earlier, the functionality of the piRNA pathway in the gonads of interspecific hybrids is potentially dependent on interspecific divergence in protein components of the pathway [51,53]. It has been found that Rhi and Del proteins in *D. melanogaster* and *D. simulans* have undergone a significant divergence owing to positive selection and form nonfunctional RDC complexes with orthologues from the alien species [51,52,63]. Ectopic expression of *D. simulans* orthologous alleles of *rhi* and *del* fails to rescue corresponding null mutations in *D. melanogaster* ovaries. *Rhi^sim^* and *del^sim^* expressed in the *D. melanogaster* germline act as dominant-negative alleles, leading to a collapse of piRNA biogenesis in the germline and causing female sterility [51]. However, it remains unknown whether these potentially epistatic interactions and defects in the functionality of RDC complexes could manifest themselves in an interspecific hybrid environment and directly contribute to hybrid sterility. Using comparative whole-transcriptome analysis, the RT-qPCR approach, and Western blot analysis, we showed that the RDC complex encoded by *D. melanogaster* alleles cannot be assembled at all in the ovarian germline of *D. mel/D. sim* interspecific hybrids (Figure 4). Despite observing a lack of expression of the *rhi^mel^* and *del^mel^* alleles in hybrid ovaries, we found that both types of major clusters inherited from the *D. melanogaster* genome, the RDC-dependent and RDC-independent ones, were still active as sources of piRNAs (Figure 5). Ping-pong signatures of TE-related piRNAs are clearly evident in the hybrid ovaries (Figure 7c), which indicates the presence of active piRNA biogenesis in the germline of hybrids. We also showed that most TEs did not exhibit a significant transcription increase in hybrids (Figure 6a). These results support the role of the RDC^sim^ complex in recognizing and licensing dual-strand piRNA clusters inherited in the hybrid genome from *D. melanogaster* mothers to provide non-canonical transcription of the clusters and subsequent piRNA biogenesis (Figure 5). Taken together, we conclude that a lack of functional RDC^mel^ complex in hybrids is at least partially compensated by the activity of the RDC^sim^ complex, independent of the origin of piRNA clusters. 

It has been shown that sequences and the TE fragment content of piRNA clusters vary significantly between species, rapidly evolving between *D. melanogaster* and *D. simulans* [68,69]. The similarity in *42AB*, the canonical dual-strand RDC-dependent piRNA cluster, between *D. simulans* and *D. melanogaster* was found to be about 4% [68]. However, piRNA clusters in the *Drosophila* genus appear to possess common properties. They have a high TE density as adaptive libraries for piRNA generation and are mainly located in heterochromatic regions of the genome that carry corresponding epigenetic marks. Rhi proteins are HP1 homologs that recognize H3K9me3 histone modification via the chromodomain [45]. However, besides the enrichment of the chromatin region by H3K9me3, other cellular and genomic factors appear to impact Rhi binding preferences. It was recently found that the DNA-binding zinc finger protein CG2678/Kipferl, together with Rhi affinity to H3K9me3, defines the majority of Rhi chromatin binding sites in the ovaries of *D. melanogaster* [70]. Assuming that the guanosine-rich relatively simple DNA motif required for Kipferl binding is conservative across *Drosophila* species genomes, we can suppose that the RDC^sim^ complex is recruited by Kipferl to a variety of piRNA clusters in the background of the hybrid genome.

In addition, we also found that the level of key protein components of the piRNA machinery, ARGONAUTE/PIWI family proteins, Aub, Piwi, and AGO3, was reduced in hybrid ovaries versus parental species (Figure 4c). Subsequent analysis of TE transcripts and TE-mapped piRNAs in the ovaries of hybrids and parental species revealed that the total TE-related piRNA pool in hybrid ovaries was from 3- to 5-fold less than in parents (Figure 7a,b), despite the fact that the ping-pong piRNA biogenesis cycle was still clearly observed for TE-mapped piRNAs in hybrid ovaries (Figure 7c). This observation allows us to propose that insufficient piRNA silencing capacity could contribute to the mobilization of TEs in the germline, causing interspecific hybrid sterility. According to several earlier published studies, an increased transcriptional level of TEs was detected in the ovaries of interspecific hybrids of *Drosophila* [32,33,35]. The genomes of *D. melanogaster* and *D. simulans* varied in their transposon content, despite sharing about 100 transposon families [65,71,72]. According to previously published data, the transcription pattern of TEs in the ovaries of hybrids between *D. melanogaster* and *D. simulans* does not reflect the presence of species-specific TEs, but TE transcript abundance and defects of biogenesis of TE-related piRNAs are similar to piRNA pathway-disrupting mutations in *D. melanogaster* [32,73]. 

It has been shown earlier that a violation of the piRNA pathway leads to subsequent developmental defects in ovaries, causing genome damage in oocyte DNA and subsequent female infertility [31,67]. Unregulated activity of TEs in hybrids could presumably be a causative factor for similar effects. By leveraging our whole-transcriptome data analysis of the main types of TEs (*LTR*, *LINE*, and *DNA*), we found that most TEs did not exhibit a significant increase in their transcriptional level in hybrid ovaries (Figure 6a). We observed that hybrid ovaries exhibited very few overexpressed TEs (Figure 6b,c). We focused on a cohort of transposons, whose expression increased more than fivefold in hybrid ovaries compared with both parental species (Figure 6c). The increasing transcriptional level of these TEs was associated with insufficient expression of TE-related piRNAs (Figure 7a,b; Table 1). Note, that all these seven TEs are residents of the *D. melanogaster* genome, and according to the crossing direction, they must have cognate maternally deposited piRNAs in hybrid ovaries. Nevertheless, our analysis revealed a reduction from 2.6- to 13.4-fold in antisense piRNAs related to these TEs in hybrid ovaries versus *D. melanogaster* ovaries (Table 1). Thus, we found that the activity of the piRNA pathway in TE regulation in hybrid ovaries was maintained at a lower level compared with both parental species. However, only two of these TEs, *blood* and *Burdock*, exhibited more than 50-fold overexpression in hybrid ovaries in comparison with both parental species (Figure 6c). It was shown that in piRNA pathway mutant ovaries of *D. melanogaster*, up to 17 TE families exhibit more than 50-fold transcriptional overexpression [64], demonstrating more stringent TE derepression compared with that in hybrid ovaries.

Could TE overexpression be considered the main reason for the disruption of the oogenesis process and hybrid sterility? It is known that two main classes of transposons, the DNA class and retrotransposons, use different strategies to carry out new insertions in the germline [31,66,74]. The vast majority of transposons do not realize their transpositions across the genome, even upon severe piRNA pathway disruption in the ovaries. Mobilization of *LINEs* and retrotransposons in germline stem cells appears to be a rare event. However, a part of the retrotransposon and *LINE* families is able to selectively integrate into the DNA of developing oocytes in egg chambers, owing to microtubule-mediated transport of their transcripts from nurse cells [31,66]. We found that a group of retrotransposons overexpressed in hybrid ovaries includes *blood*, *rover*, *HMS-Beagle*, and *diver*, which can potentially be inserted into the genome of oocytes. Among them, *HMS-Beagle* and *blood* have been found among the top four active transposons, which cause 91% of the total insertions detected in oocyte DNA in *D. melanogaster* with double AGO3 and Aub depletion [31,66]. However, our analysis revealed the presence of DSBs (traces of insertions by the “cut-and-paste” mechanism) in the karyosomes of hybrid ovaries only in 23.5% of the cases, indicating that increased activity of retrotransposons did not constitute a major cause of hybrid female sterility (Figure 2c,f). We assumed that illegitimate TE mobilization can contribute to some extent to oogenesis violations, but it is not the major reason for the female sterility of *D. mel/D. sim* hybrids. 

A comprehensive analysis of TE regulation in the gonads of interspecific hybrids of two sibling species, *Drosophila arizonae* and *Drosophila mojavensis wrigleyi*, members of the *repleta* group within the *Drosophila* genus, with a divergence time of around 1.5 MYA was carried out in the recent paper [34]. These investigations show that in hybrid gonads, compared with both parental species, very few TEs are derepressed. The interspecies divergence of TE copies and a reduced repertoire of specific TE-related piRNAs are shown to be associated with their derepression. These results also show that no genes implicated in the piRNA pathways show DE in the hybrid ovaries when compared with the parental species [34]. In our study, we also found the absence of massive TE expression and mobilization in the ovaries of hybrids between the species with an estimated divergence time of about three MYA [15,16,17]. This resembles a situation describing interspecific hybrids between *Drosophila arizonae* and *Drosophila mojavensis wrigleyi*, which split relatively recently [34], but not the studies that underscore the significant impact of TE mobilization in causing interspecific hybrid sterility in the *Drosophila* genus [33,35]. However, unlike data from Banho and coworkers [34], we found differential expression of parental alleles encoding essential piRNA pathway components in the ovaries of interspecific hybrids (Figure 4).

It is known that RNA helicase Vasa is a conservative germline marker required for piRNA biogenesis and transposon silencing in *Drosophila* [39,75,76]. Atrophy of the germarium, loss of GSCs, cystoblasts, dividing cysts, and degeneration of egg chambers in middle oogenesis often occur in fly females with *vasa* null mutations [76,77]. Because premature GSC loss was found to be the most prominent defect of oogenesis in hybrid ovaries, we decided to study the pattern of Vasa expression in hybrid ovaries. Taking into account earlier published data about allele-specific silencing of *D. mauritiana vasa* by *AT-chX* piRNAs originated from the *D. melanogaster* genome and its contribution to defects of early spermatogenesis of hybrid *D.mel/D.mau* males [11], we analyzed relationships between *AT-chX* piRNAs and *vasa* expression in *D. mel/D. sim* hybrid ovaries. We confirmed that the *AT-chX* piRNA cluster was active in the ovaries of *D. mel/D. sim* hybrids, providing both sense and antisense piRNAs (Figure 8a). As expected, we found clear ping-pong signatures only between *AT-chX*- and *vasa^sim^*-mapped piRNA pairs but not for *vasa^mel^* (Figure 8b). According to the presence of *AT-chX* piRNAs, we detected a significantly lowered level of Vasa^sim^ protein in hybrid ovaries (Figure 8d). This indicates that epigenetic factors, *AT-chX* piRNAs, impact the expression regulation of the critically important oogenesis gene. In addition, we also uncovered that zygotic expression of the Vasa^mel^ protein occurred only in middle oogenesis with a significant developmental delay (Figure 8c). Considering multiple defects of the early stages of oogenesis in hybrid ovaries, we suppose that a deficiency of Vasa dose in the hybrid germline could contribute to observed female hybrid sterility manifestations, including a premature loss of GSCs and subsequent oogenesis defects. However, this assumption needs further investigation.

Gametogenesis is often disrupted in the progeny of interspecific crosses; however, underlying mechanistic causes and factors remain poorly understood. We observed that violations of piRNA-mediated TE regulation in the ovaries of *D. mel/D. sim* hybrid females did not lead to massive transposon invasions in the hybrid genome but could contribute in part to the genome disintegration of developing oocytes. At the same time, piRNA targeting of *vasa^sim^* transcripts in the germline of hybrid ovaries, together with a delay of zygotic expression of *vasa^mel^*, appears to underlie a wide range of oogenesis defects. We conclude that the hybrid sterility phenotype is based on complex multi-locus genetic and epigenetic divergence between these sibling species.

## 4. Materials and Methods

### 4.1. Fly Stocks and Genetics

To analyze the hybrid sterility in crossings between *D. melanogaster* and *D. simulans* flies, we used the *Hmr^2^* line of *D. melanogaster* from the Bloomington Drosophila Stock Center (*In(1)w[m4]*, *In(1)AB*, *y[2] Hmr[2]*, # 1304). This line carries the *In(1)AB* inversion, which provides a translocation of the 359 bp *1.688* satellite megablock out of the pericentric heterochromatin of the X chromosome [18,78]. It was shown that the presence of this 359 bp satellite block in the pericentric area induces failures in the correct segregation of paternally inherited X^mel^ chromosomes in hybrid embryos. Mutations in the *Hmr* gene are shown to rescue F1 hybrid progeny in crosses of *D. melanogaster* with sibling species of the *Simulans* clade [7,32]. Thus, two rescue activities are reported for this *D. melanogaster* line. *Drosophila simulans* stock *w.501* was obtained from the UC San Diego *Drosophila Species Stock Center*. For crossing, we put *Hmr^2^* *D. melanogaster* virgin females in vials containing 10 mL of a standard laboratory medium (from 8 to 10 females per vial) with twice as many very young *w.501 D. simulans* males. F1 interspecific hybrid females from the cross of *Hmr^2^* females to *w.501* males were collected from crosses at 20 °C and reared at 20 °C on a standard cornmeal medium. The flies were subjected to a laboratory light–dark cycle of 12:12 h. Offspring hybrid females were aged up to 3–6 days after eclosion.

### 4.2. Immunofluorescence Staining and Confocal Microscopy

Ovaries of adult females were manually dissected in phosphate-buffered saline (PBS) at 4 °C, washed with PBT (1 × PBS, 0.1% Tween 20), and fixed in 3.7% formaldehyde and PBT for 30 min at room temperature. All the following procedures were carried out as described previously [79]. Staining was detected by laser scanning confocal microscopy using a Carl Zeiss LSM 510 META machine (Carl Zeiss, Oberkochen, DE, Germany). All images were taken with a z-resolution of 0.5–1 µm. The obtained pictures were imported into Imaris^®^ 5.0.1 (Bitplane AG, Belfast, UK) for subsequent processing. The data were analyzed using Excel 2016 (https://www.microsoft.com/ru-ru/microsoft-365/previous-versions/microsoft-office-2016) and RStudio (https://www.r-project.org/).

### 4.3. Antibodies

The following antibodies were used for immunofluorescence staining: a mix of murine monoclonal anti-Lamin Dm0 ADL67.10 and ADL84.12 antibodies (Developmental Studies Hybridoma Bank, University of Iowa (DSHB), IA, USA), 1:500; rabbit polyclonal anti-Lamin antibodies [80], 1:500; rat monoclonal anti-Vasa antibody (DSHB), 1:100; guinea pig polyclonal anti-Trafic jam (Tj) antibodies [81], 1:5000; murine monoclonal anti-Traffic jam (Tj) antibody [82], 1:100; rabbit polyclonal anti-Vasa antibodies (gift of R. Lehmann), 1:500; rabbit polyclonal anti-γH2Av (pS137) antibodies (Rockland), 1:500; and murine monoclonal anti-Orb (4H8, DSHB) antibody, 1:150. Alexa Fluor-labeled secondary goat anti-rat IgG, goat anti-rabbit IgG, goat anti-mouse IgG, and goat anti-guinea pig IgG (Invitrogen, Waltham, MA, USA) were used as secondary reagents at a dilution of 1:500. DAPI (40,6-diamidino-2-phenylindole) (Sigma, St. Louis, MO, USA) was used for chromatin staining.

The following antibodies were used for Western blot analysis: murine monoclonal anti-β-actin antibody ab8224 (Abcam, Cambridge, UK), 1:4000; rat monoclonal anti-Vasa antibody (DSHB), 1:4000; rabbit polyclonal anti-Vasa antibodies (R. Lehmann gift), 1:5000; murine monoclonal anti-AGO3 9G3 antibody [38], 1:250; murine monoclonal anti-Aub 4D10 antibody [83], 1:500; rabbit polyclonal anti-Piwi 2464 antibodies [37], 1:2000; rat polyclonal anti-Rhino antibodies [84], 1:500; and murine monoclonal anti-Cutoff antibody 9D5 (DSHB), 1:130. Samples were resolved by SDS-PAGE and blotted onto PVDF membrane Immobilon-P (Sigma). Alkaline phosphatase-conjugated anti-mouse, anti-rabbit, and anti-rat antibodies (Sigma) were used as secondary reagents at a dilution of 1:20,000. Blots were developed using the Immun-Star AP detection system (Bio-Rad Laboratories, Hercules, CA, USA). All experiments were performed at least in triplicate with independent preparations of ovarian lysates. See Figure S5 for full original images of immunoblots that presented in the figures.

### 4.4. RT-qPCR Analysis

For RT-qPCR analysis, total RNA was isolated from 30 to 50 pairs of dissected ovaries using TRIzol Reagent (Invitrogen). Complementary DNA (cDNA) was synthesized using random hexamers and SuperScriptII reverse transcriptase (Invitrogen). cDNA samples were analyzed by PCR or real-time quantitative PCR using the incorporation of SYTO13 (Invitrogen). All experiments were performed with at least three independent RNA samples; each sample was analyzed in duplicate. Fold change ratios of the average expression level were calculated. The Fieller method was used to estimate confidence intervals. We used *rp49* (*rpL32*) as a loading control. The following primers were used for RT-PCR: *rp49* fw 5′-ATGACCATCCGCCCAGCATAC-3′, rev 5′-GCTTAGCATATCGATCCGACTGG-3′; *piwi* of *D. melanogaster* dmel_piwi_2ex fw 5′-CCACGGCTTTCGCTCTGTC-3′, rev 5′-CCGAGAGCGGATCCTCGTAC-3′; *piwi* of *D. simulans* dsim_piwi_2ex fw 5′-CACGGCCTTCGCTCTGGT-3′, rev 5′-CCGAGAGTGGATCCTCGAG-3′; *aub* of *D. melanogaster* aub_dmel_1 fw 5′-CATGAGTGAACATACCAGGCTGAA-3′, rev 5′-GCGGAGTCCAGCTCGATGTT-3′; *aub* of *D. simulans* aub_dsim_1 fw 5′-ATGAATGAACATACCAGGAGCCC-3′, rev 5′-GCGGTGTCAAGCTCGATGTTC-3′; *vasa* of *D. melanogaster* vasa_mel_5ex fw 5′-GAGCTTGGAAGACCCCAGGTAGTG-3′, rev 5′-GTCTTCAAACGTGATAAAGGTCCGAT-3′; *vasa* of *D. simulans* vasa_sim_5ex fw 5′-CCGCAGGCAGTGATTGTATCC-3′, rev 5′-GTCTTCAAAGGTGACAAAAGTCCGAT-3′; *rhino (rhi)* of *D. melanogaster* rhi_1ex_dmel fw 5′-CGGTTTTCCGAACGAGAAC-3′, rev 5′-CGGCCTTCCGATGCA-3′; *rhino (rhi)* of *D. simulans* rhi_1ex_dsim fw 5′-CCAAGGCTCCACAGGCTT-3′, rev 5′-CCACAGCTTCGTCTTCGC-3′; *cutoff (cuff)* of *D. melanogaster* dmel_cuff_1ex fw 5′-CTGGATGGTCATTGGGTGTC-3′, rev 5′-CCTCTTCAGATTGTCCTGATCAAT-3′; *cutoff (cuff)* of *D. simulans* dsim_cuff_1ex fw 5′-CTGGATGGTCTCTGGGTGTT-3′, rev 5′-ACCCTCTTCAGATTGTCTTCAGTAAA-3′; and *deadlock (del)* of *D. melanogaster* del_mel1 fw 5′-GGTCCACACTTCCAGAGTCC-3′, rev 5′-AGGATCACAACCTGTTGAACCT-3′, *deadlock (del)* of *D. simulans* del_sim1 5′-GGCCACACCTCCAGAGTC-3′, rev 5′-CCATCACCTGTTGAATTTACTGAG-3′.

### 4.5. RNA Library Preparation

For the preparation of RNA-seq libraries, RNA was isolated from *D. melanogaster Hmr^2^*, *D. simulans w501*, and interspecific hybrid ovaries with TRIzol Reagent (Invitrogen). PolyA selection was performed using a Dynabeads™ mRNA Purification Kit (Thermo Fisher Scientific, Waltham, MA, USA). RNA-seq libraries were generated using the NEBNext Ultra™ II Directional RNA Library Prep Kit for Illumina (#E7760, NEB, UK) and NEBNext Multiplex Oligos for Illumina (Index Primers Set 1, NEB #E7335, and Set 2, NEB #E7500) according to the manufacturer’s instructions and sequenced on the Illumina NovaSeq 6000 SP platform (100 bp runs) in Evrogen. For small RNA library preparation, RNA was extracted from 0–3 days old gonads of *D. melanogaster Hmr^2^*, *D. simulans w501*, and interspecific hybrids. Small RNA fraction (19–29 nt) was purified by 15% polyacrylamide gel from 20 to 30 μg total RNA. Libraries were prepared using NEBNext Multiplex Small RNA Sample Prep Set for Illumina (E7300S) and sequenced on NovaSeq 6000 in 100 bp single-end mode yielding 25–40 million single-end reads per library. All library types were prepared and sequenced in two biological replicates. The GEO accession number for the library data is GSE263985.

### 4.6. Bioinformatics Analysis

Quality control of the sequenced libraries was carried out using the FastQC tool. For subsequent analysis, only reads with fastq score > 30 were used. RNA-seq libraries of the ovaries of hybrids and parental species were pseudo-aligned by kallisto v.0.50.0 [85] with the following parameters: --single, -l 100, and -s 0.05. Merged transcriptome sets obtained from NCBI were used as a reference (GCF_000001215.4 and GCF_016746395.2 assemblies for *D. melanogaster* and *D. simulans*, respectively). Quantified abundances of transcripts for protein-coding genes were imported with tximport v.1.24.0 [86]. In the case of parental species, transcript abundance was represented only by species-specific transcripts (more than 95% of reads), while reads were equally distributed between species-specific transcriptome sets in the case of hybrids. For TE expression analysis, transcriptomic polyA libraries were trimmed using cutadapt 2.8 and mapped to *Drosophila* transposon canonical sequences v10.1 (https://github.com/bergmanlab/drosophila-transposons/ (accessed on 30 August 2021)) using bowtie version 1.1.0 with parameters -l 100, -v 3, and -m 1. The read-count step htseq-count 2.0.0 was used. 

Differential expression analysis was performed using the R package DESeq2 v.1.36.0 [87]. Genes are considered to be differentially expressed when they present two-fold differences and FDR-corrected *p*-values < 0.05. The GO analysis was performed using ShiniGO 0.80 software [61]. For subsequent analysis of allele-specific gene expression in hybrid gonads, we identified orthologous genes in the *D*. *melanogaster* and *D. simulans* genomes. A set of protein-coding gene transcripts from *D. melanogaster* was analyzed using BLAST against transcripts from *D. simulans* retrieved from the RefSeq database (https://ftp.ncbi.nlm.nih.gov/refseq/release/ (accessed on 11 May 2020)), and the top hits with the highest bitscore were selected as homologous in *D. simulans*. We considered genes as orthologous if all transcripts from *D. simulans* locus corresponded to the same gene in *D. melanogaster*. Using this approach, more than 90% of the *D. melanogaster* genes were identified as orthologues with *D. simulans* genes. The piRNA pathway genes were further manually curated. 

Differential expression of TE families was made independently between parental lines and between hybrids and parental lines. TE families were classified as differentially expressed when |log2(FC)| > 2.32 (fivefold difference) and FDR-corrected *p*-values < 0.05. For piRNA production analysis, adapter sequences were removed by cutadapt 2.8. After quality control with FastQC, rRNA, snRNA, snoRNA, and tRNA were filtered from the libraries using bowtie (version 1.1.0) with the -v 3 parameter. Unaligned reads were then filtered against the microRNA sets of *D. simulans* and *D. melanogaster* obtained from https://ftp.flybase.net/releases/current/dsim_r2.02/ (accessed on 16 June 2020) and (accessed on 22 February 2024), respectively. Small piRNAs of 23–29 nt in length were selected using cutadapt 2.8. This piRNA fraction was mapped to *Drosophila* transposon canonical sequences v10.1 (https://github.com/bergmanlab/drosophila-transposons/ (accessed on 30 August 2021)) using bowtie version 1.1.0 with parameters -l 35 and -n 3. Mapped reads were counted with htseq-count 2.0.0 and normalized to the library depth before microRNA filtration. To analyze the functionality of the piRNA pathway, ping-pong signatures for each TE family were calculated using a signature.py script [88].

To analyze the activity of the piRNA clusters of *D. melanogaster*, piRNAs were uniquely mapped to a merged file containing both the *D. melanogaster* and *D. simulans* genomes using bowtie (-l 50, -v 0, and -m 1). The read count step was made by htseq-count 2.0.0. For this, we used the cluster coordinates obtained earlier [64]. The counted values were normalized to the library depth before microRNA filtering, as in the case of transposon piRNA analysis. To analyze the production of piRNAs from the *AT-chX* cluster, piRNAs were mapped to the consensus *AT-chX* sequence obtained previously [11] using bowtie (-l 50, -n 3). The counting of mapped piRNAs was performed using htseq-count 2.0.0. The distribution density of reads in clusters was visualized using pyGenomeTracks 3.8 [89]. Ping-pong signature analysis of mapped reads was carried out by a signature.py script [88]. Most data visualization and analysis were performed in Python 3 using pandas (https://zenodo.org/records/10697587 (accessed on 23 February 2024)), seaborn [90], numpy [91], and matplotlib [92] software packages.

## Figures and Tables

**Table 1:** Estimation of piRNA silencing potential for seven derepressed TEs in parental and hybrid ovaries. Silencing potential is considered to be true if there are >100 rpm antisense TE-mapped piRNAs (0–3 mm), according to [64]. For four overexpressed TEs, silencing potential in hybrid ovaries was not found to be enough.

TE Family	TE-Mapped Antisense piRNAs in *D. sim* Ovaries, rpm	TE-Mapped Antisense piRNAs in *D. mel* Ovaries, rpm	TE-Mapped Antisense piRNAs in Hybrid Ovaries, rpm
*blood*	5602.2	2904.2	1112.3
*Burdock*	627.6	1072.6	80.3
*gypsy12*	1574.0	1011.6	131.7
*diver*	1163.3	424.4	52.6
*HMS-Beagle*	1834.4	407.8	89.5
*rover*	51.0	1335.5	362.7
*jockey*	182.3	375.3	45.6

## Data Availability

The library datasets used for analysis in the current study were placed in GEO with the accession number GSE263985.

## Supplementary Materials

The supporting information can be downloaded at: https://www.mdpi.com/article/10.3390/ijms25115681/s1.
