# Supplementary material for: Molecular Insights into Female Hybrid Sterility in Interspecific Crosses between Drosophila melanogaster and Drosophila simulans
Source: Int J Mol Sci. 2024 May 23;25(11):5681. doi: 10.3390/ijms25115681 (PMC11172174; doi:10.3390/ijms25115681)
Supplement: Supplementary file 1 [file ijms-25-05681-s001.zip › Supplementary Figures S1-S5.pdf]

Supplementary information of research article

Molecular insights into female hybrid sterility in interspecific crosses between *Drosophila melanogaster* and *Drosophila simulans*

Alexei A. Kotov†, Vladimir E. Adashev†, Ilia A. Kombarov, Sergei S. Bazylev, Aleksei S. Shatskikh, Ludmila V. Olenina*

The file contains Figures S1-S5 with figure legends

Figure S1

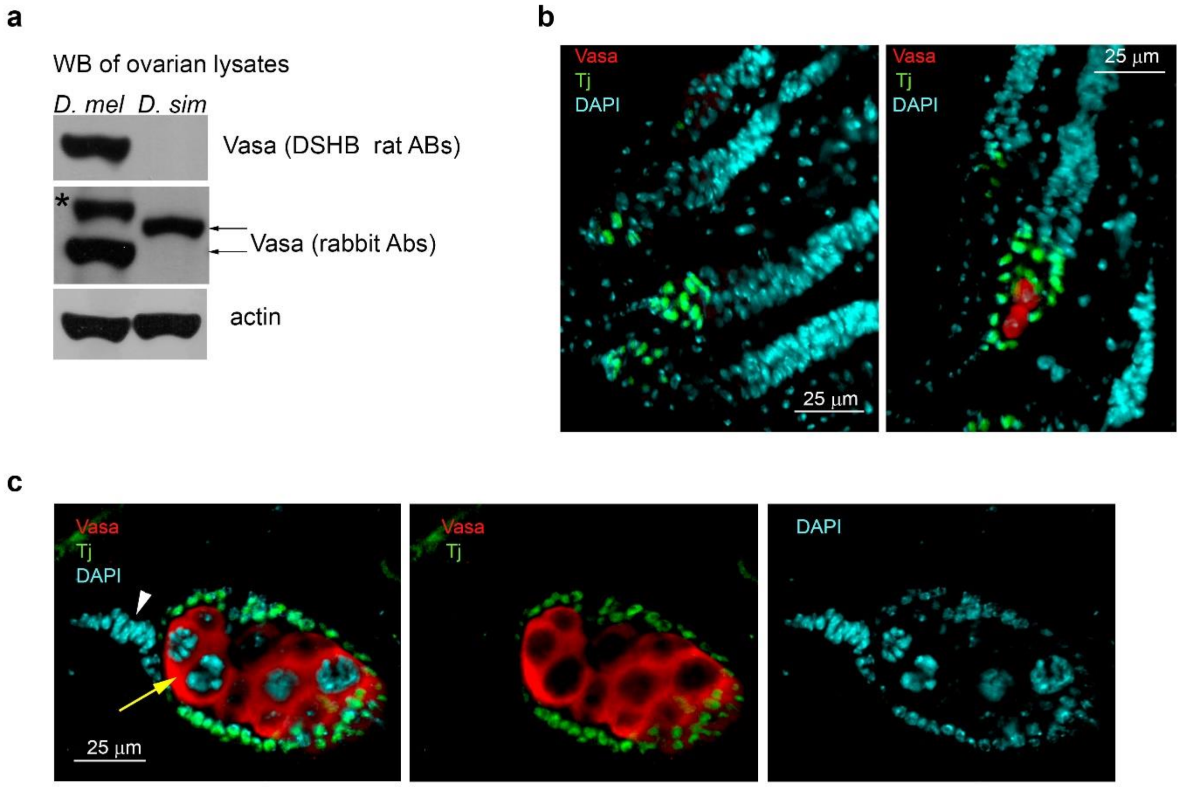

Figure S1. Immunochemical and immunofluorescence detection of germ cells in the ovaries. (**a**) Western blot analysis of ovarian lysates of *D. melanogaster* and *D. simulans* females revealed differential specificity of anti-Vasa antibodies. The rat monoclonal antibody (DSHB) is able to recognize only the Vasa protein encoded by *D. melanogaster* genome. The rabbit polyclonal antibodies recognize both orthologous Vasa proteins (arrows), Vasa^{mel} and Vasa^{sim}, with similar efficiency. * indicates an unspecified band. Anti-actin antibodies were used for loading control. (**b, c**) Immunofluorescence analysis of hybrid ovaries. Fixed ovarian preparations were stained with rabbit polyclonal antibodies to Vasa (germ cell marker, red) and Tj (marker of somatic ovarian cells, green), chromatin was stained with DAPI (blue). The majority of germariums of hybrid ovaries did not contain germ cells. However, some ovarioles in the hybrid ovaries contained from one to multiply Vasa-positive germ cells (**b**). (**c**) The presence of differentiating germ cells in the apical part of the germarium of hybrid ovary, where GSCs should normally be located. The yellow arrow indicates a developing cyst of germ cells with large nuclei. The white arrowhead indicates terminal filament cells at the anterior tip of the germarium.

Figure S2

hybrid oocytes

oocytes of

D. melanogaster

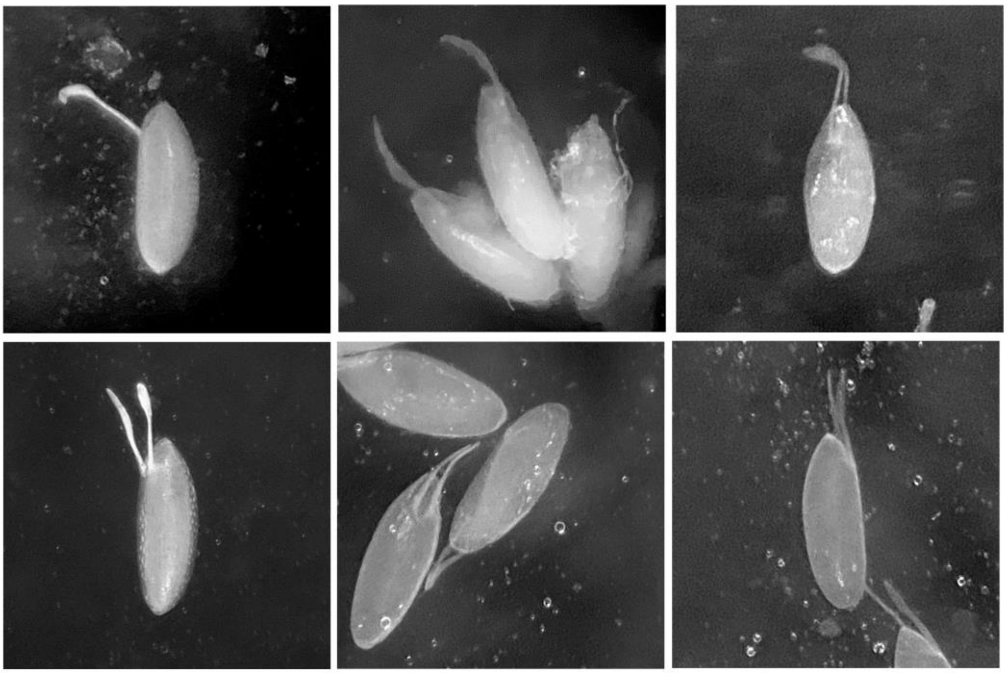

Figure S2. Dorsal appendage defects in hybrid oocytes. Mature *D. melanogaster*/*D. simulans* hybrid oocytes (top row of images) have fused dorsal appendages in 69.1% (n = 68) of cases, indicating defects of the egg dorsoventral patterning. Mature oocytes of *D. melanogaster* Hmr^2 (bottom row of images) generally exhibit (n = 32) the presence of two separated dorsal appendages at the anterior end.

Figure S3

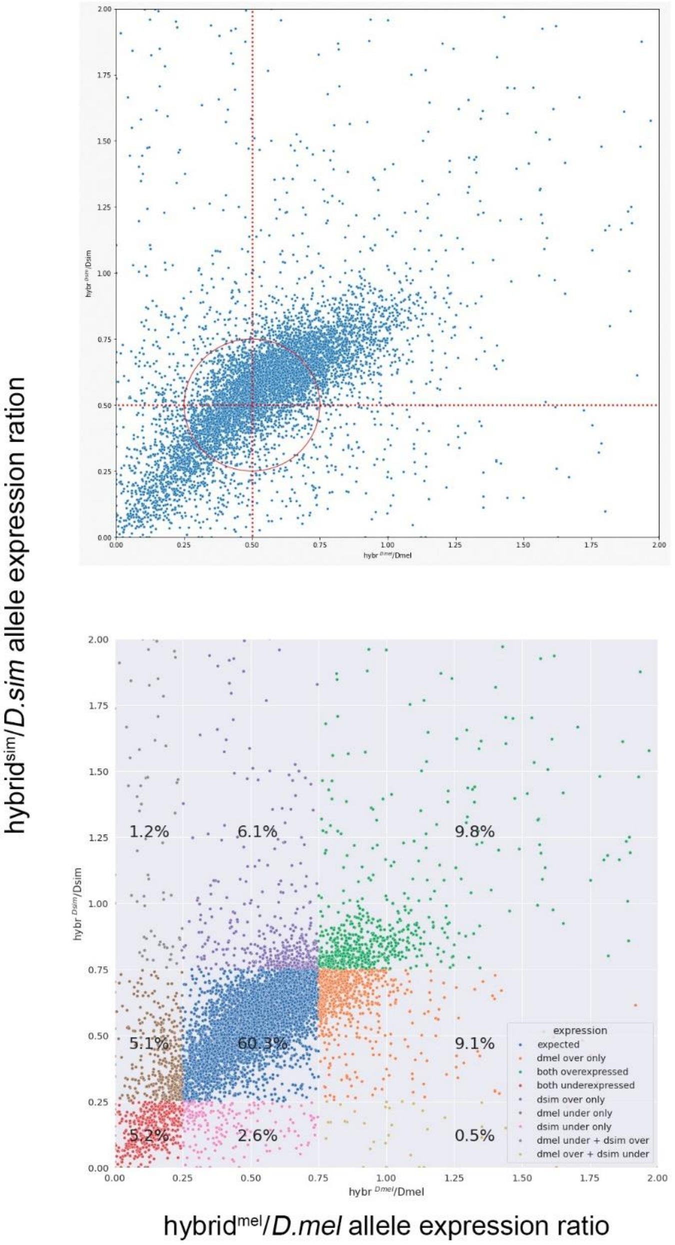

Figure S3. Analysis of the expression level of *D. melanogaster* and *D. simulans* inherited genes in the hybrid ovaries relative to their expression in the parental species. The scatterplot represents a comparison of hybrid/parent expression ratios for species-specific orthologues. The top image presents the scatterplot with red dotted lines indicating half-dose levels, and the region contained genes with expected expression of both orthologous alleles (red dotted circle). The bottom image contains the dot color marks that indicate the comparative categories of genes in hybrid ovaries with similar expression patterns as indicated in the legend on the graph. See also Table S3 for additional data.

Figure S4

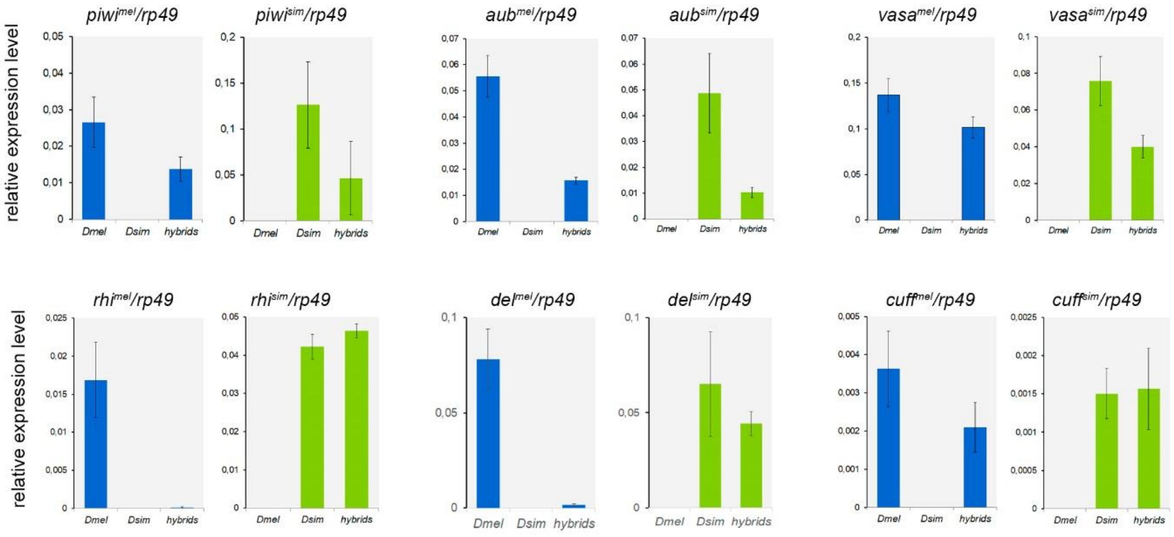

Figure S4. RT-qPCR analysis of piRNA pathway gene expression in the ovaries of hybrids compared to parental species with allele-specific primers. Bar colors indicate the relative transcriptional level of species-specific alleles in hybrids: blue for *D. melanogaster* and green for *D. simulans*. The expression levels of mRNAs are normalized to *rp49* transcripts. Data from three independent experiments are shown as average values with standard deviations. In accordance with the results of the DEseq analysis, we observed the absence of *rhi*^{mel} and *del*^{mel} allele expression.

Figure S5a

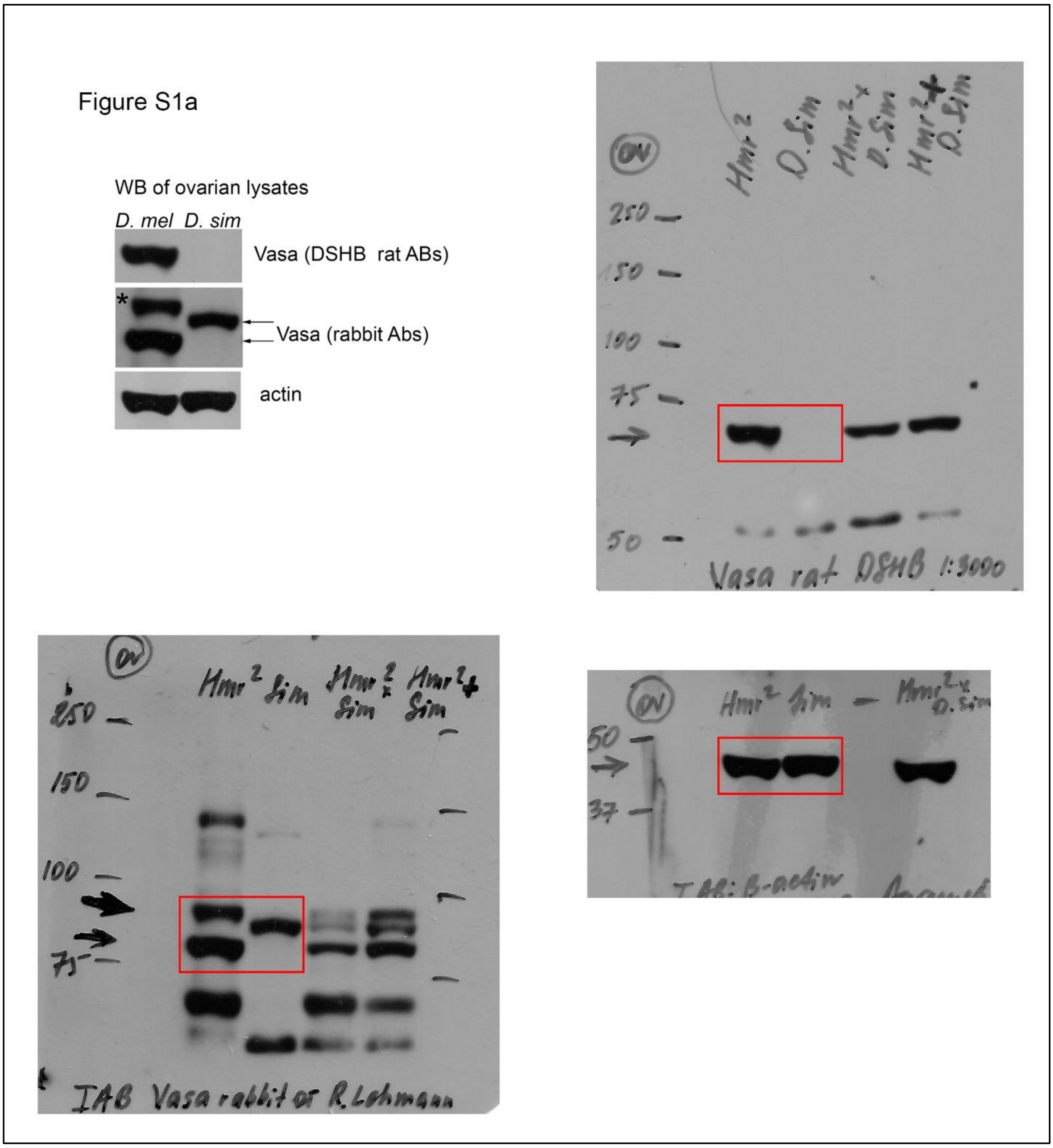
Figure S1a
WB of ovarian lysates
D. mel D. sim
Vasa (DSHB rat ABs)
Vasa (rabbit Abs)
actin
Vasa rat DSHB 1:3000
250
150
100
75
50
IAB Vasa rabbit of R. Lehmann
37

FigureS5b

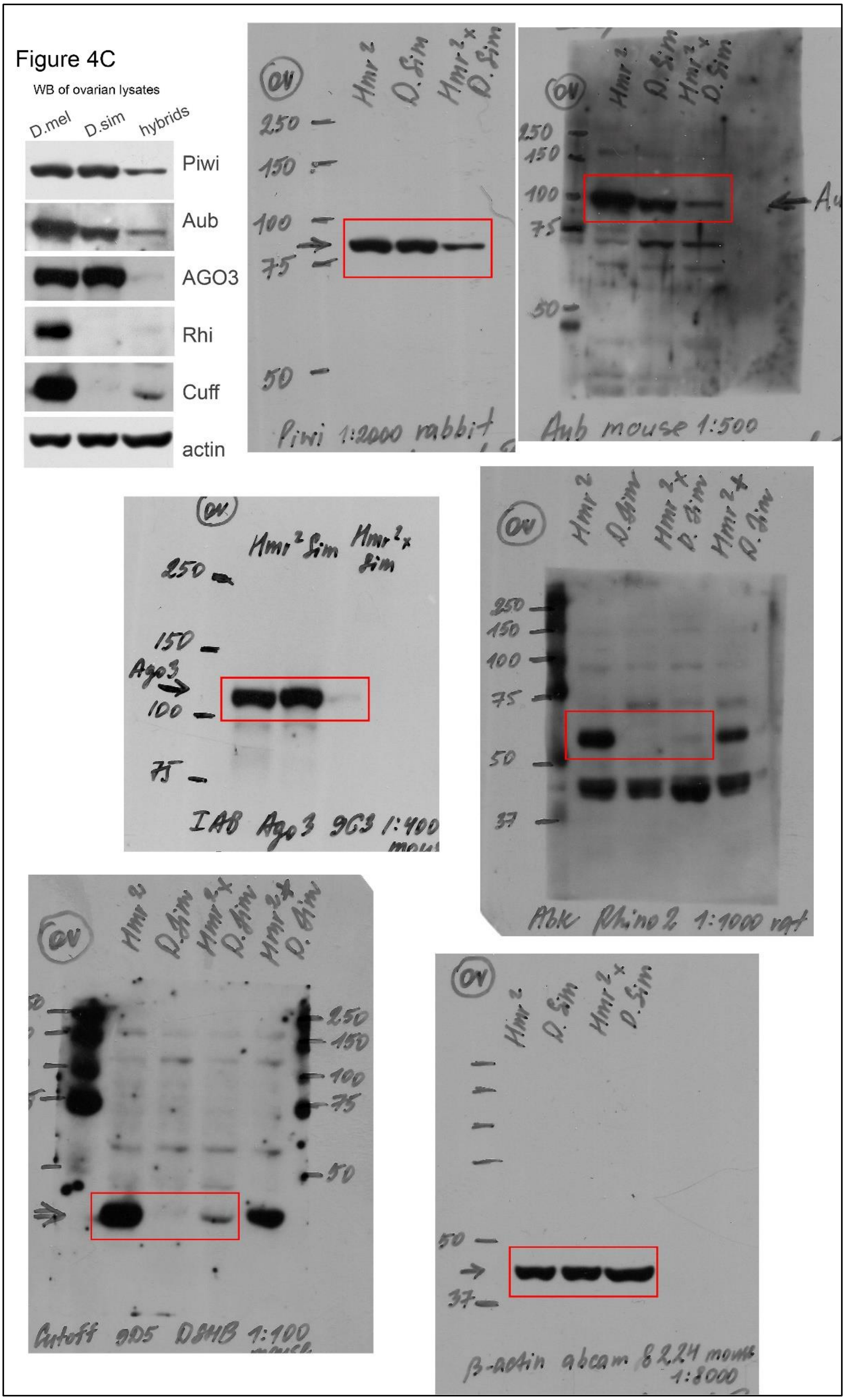

Figure 4C
WB of ovarian lysates
D.mel
D.sim
hybrids
Piwi
Aub
AGO3
Rhi
Cuff
actin

Figure S5c

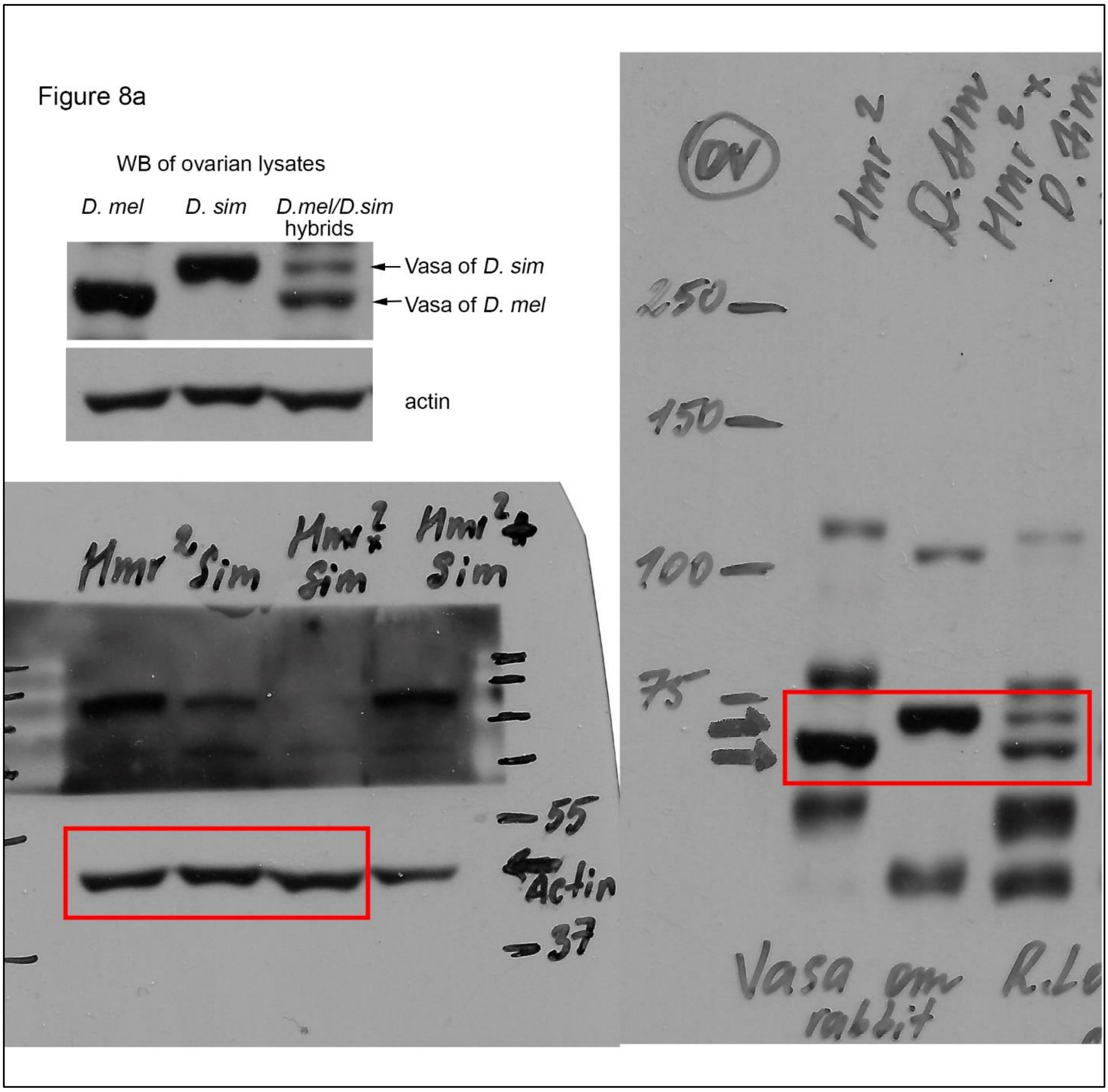

Figure S5. Full original images of immunoblots which presented in the article. Markers of molecular weight are indicated on the side. Images displayed as parts of the figures are shown at the top left of each panel. (**a**) Original images for Figure S1a. (**b**) Original images for Figure 4c. (**c**) Original images for Figure 8a.
